# Clinical impact of pharmacogenetic profiling with a clinical decision support tool in polypharmacy home health patients: A prospective pilot randomized controlled trial

**DOI:** 10.1371/journal.pone.0170905

**Published:** 2017-02-02

**Authors:** Lindsay S. Elliott, John C. Henderson, Moni B. Neradilek, Nicolas A. Moyer, Kristine C. Ashcraft, Ranjit K. Thirumaran

**Affiliations:** 1 Department of Pharmacy Practice, Harding University College of Pharmacy / Unity Health – White County Medical Center, Searcy, Arkansas, United States of America; 2 Unity Health - White County Medical Center, Searcy, Arkansas, United States of America; 3 The Mountain-Whisper-Light Statistics, Seattle, Washington, United States of America; 4 Clinical Pharmacogenomics Division, Genelex Corporation, Seattle, Washington, United States of America; University Hospital Jena, GERMANY

## Abstract

**Background:**

In polypharmacy patients under home health management, pharmacogenetic testing coupled with guidance from a clinical decision support tool (CDST) on reducing drug, gene, and cumulative interaction risk may provide valuable insights in prescription drug treatment, reducing re-hospitalization and emergency department (ED) visits. We assessed the clinical impact of pharmacogenetic profiling integrating binary and cumulative drug and gene interaction warnings on home health polypharmacy patients.

**Methods and findings:**

This prospective, open-label, randomized controlled trial was conducted at one hospital-based home health agency between February 2015 and February 2016. Recruitment came from patient referrals to home health at hospital discharge. Eligible patients were aged 50 years and older and taking or initiating treatment with medications with potential or significant drug-gene-based interactions. Subjects (n = 110) were randomized to pharmacogenetic profiling (n = 57). The study pharmacist reviewed drug-drug, drug-gene, and cumulative drug and/or gene interactions using the YouScript^®^ CDST to provide drug therapy recommendations to clinicians. The control group (n = 53) received treatment as usual including pharmacist guided medication management using a standard drug information resource. The primary outcome measure was the number of re-hospitalizations and ED visits at 30 and 60 days after discharge from the hospital.

The mean number of re-hospitalizations per patient in the tested vs. untested group was 0.25 vs. 0.38 at 30 days (relative risk (RR), 0.65; 95% confidence interval (CI), 0.32–1.28; P = 0.21) and 0.33 vs. 0.70 at 60 days following enrollment (RR, 0.48; 95% CI, 0.27–0.82; P = 0.007). The mean number of ED visits per patient in the tested vs. untested group was 0.25 vs. 0.40 at 30 days (RR, 0.62; 95% CI, 0.31–1.21; P = 0.16) and 0.39 vs. 0.66 at 60 days (RR, 0.58; 95% CI, 0.34–0.99; P = 0.045). Differences in composite outcomes at 60 days (exploratory endpoints) were also found. Of the total 124 drug therapy recommendations passed on to clinicians, 96 (77%) were followed. These findings should be verified with additional prospective confirmatory studies involving real-world applications in larger populations to broaden acceptance in routine clinical practice.

**Conclusions:**

Pharmacogenetic testing of polypharmacy patients aged 50 and older, supported by an appropriate CDST, considerably reduced re-hospitalizations and ED visits at 60 days following enrollment resulting in potential health resource utilization savings and improved healthcare.

**Trial registration:**

ClinicalTrials.gov NCT02378220

## Introduction

In 2013, Home Health Agencies (HHAs) provided services to about 3.5 million Medicare beneficiaries, and Medicare spent about $18 billion on home health services [1]. Most patients receiving home health care are elderly, take multiple medications, and experience poor compliance due to a number of issues, primarily adverse drug events (ADEs). This often leads to emergency department (ED) visits, re-hospitalizations, and decreased quality of life. With trends toward bundling of post-inpatient services, penalties for readmissions, and integration into Accountable Care Organizations, there is increased interest in hospitals co-managing or even acquiring home health providers. There is hope that innovative medical management can improve outcomes and reduce costs.

Medication-related problems are defined as circumstances during drug treatment that actually or potentially interfere with optimal care outcomes [2]. They are common among polypharmacy patients taking multiple medications and can cause adverse drug reactions (ADRs), which are adverse drug events (ADEs) at normal doses for approved uses. For example, routinely prescribed psychiatric medications are a common cause of ADR-driven ED visits [3]. Pharmacogenetic variation also leads to ADRs, including single drug-gene interactions (DGIs) and cumulative drug-drug-gene Interactions (DDGIs). More than 85% of patients have significant genetic variation in the cytochrome P-450 (CYP 450) genes that metabolize the majority of the most commonly prescribed medications [4, 5]. Genetic variance among patients who are abnormal metabolizers compounds the potential risk for ADRs and often results in decreased efficacy [6, 7]. Both drug-drug and drug-gene medication related problems rise with polypharmacy, and two-thirds of adults over age 65 use one or more prescription drugs daily [8–10]. An estimated 35% of seniors experience ADEs, nearly half of these preventable [10] and 10–17% of hospitalizations of older patients are directly related to ADRs [11]. Additionally, patients aged 60 years and older account for 51% of ADR-related deaths [12, 13].

Pharmacogenetic testing has the potential to predict and reduce unnecessary drug-related adverse reactions and its value has been shown in clinical trials in psychiatry [14]. Based on these results, we believe that adding pharmacogenetic and cumulative information warnings to drug interaction clinical decision support tools (CDST) available to pharmacists could be useful in managing polypharmacy elderly patients. Interactions involving genes cause approximately 47% (25% were DGIs and 22% were DDGIs) of significant interaction warnings that can lead to side effects and ADRs [15, 16].

Knowledge of a patient’s pharmacogenetic profile and gene-based ADR susceptibility allows clinicians to make medication therapy decisions specific to each patient leading to better outcomes such as decreased pill burden, improvement of disease state, decreased ADRs, and increased quality of life. However, controlled trials of integrated pharmacogenetic information in a broad polypharmacy population have been lacking. Pharmacogenetic profiling reduced hospitalizations (39%) and ED visits (71%) in a prospective registry study in an ambulatory Medicare-age polypharmacy population [17]. Therefore, we undertook a randomized controlled trial in a high-risk population of chronically ill patients aged 50 years and older admitted to home health care after an inpatient hospitalization. The trial was designed to add pharmacogenetic data in an integrated clinical information system compared to a standard drug information system. In addition to re-hospitalization and ED visit metrics; we examined secondary endpoints including death and Medicare administrative quality measures.

## Methods

The home health trial was approved by the Harding University Institutional Review Board (IRB) and conducted in accordance with the International Conference on Harmonization guidelines for Good Clinical Practice (ICH E6), and the Code of Federal Regulations on the Protection of Human Subjects (45 CFR Part 46) and is registered with ClinicalTrials.gov, number NCT02378220. Written informed consent was obtained from all participants.

### Setting, study population and recruitment

This pilot study was conducted at a hospital-based HHA in Searcy, Arkansas. The study population was derived from patient referrals to home health upon hospital discharge. A pharmacist and/or pharmacy intern performed chart reviews for every new patient admitted to home health and the study investigator screened potential candidates from this allotment of patients. Inclusion criteria were patients aged 50 years and older taking or initiating treatment with at least one of fifty-five single ingredient or six medication combinations [17] with potential for significant gene-based interactions defined by FDA boxed warning, FDA cautionary labeling, clinical literature, or a YouScript^®^ CDST algorithm-predicted significant effect.

**Fig 1:**
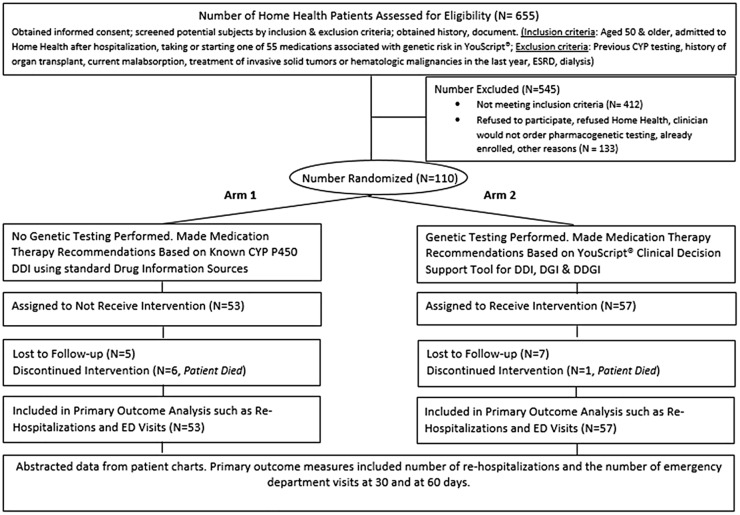
Patient selection flow chart. Of 655 home health patients assessed for eligibility, 412 did not meet the inclusion criteria and 133 patients either declined to participate, refused home health, already enrolled or the clinician would not order pharmacogenetic testing. Of 110 randomly allocated patients, 53 patients in arm 1 were assigned to not receive intervention and 57 patients from arm 2 were assigned to received pharmacogenetic intervention. CYP, cytochrome; DDI, drug-drug interaction; DGI, drug-gene Interaction; DDGI, drug-drug-gene interaction; ED, emergency department; ESRD, end stage renal disease; N, number of patients.

Exclusion criteria were the same for tested and untested groups and included patients previously tested for CYP 450, history of organ transplant, current malabsorption, treatment of invasive solid tumors or hematologic malignancies in the last year, end stage renal disease or current dialysis. Of 655 patients assessed for eligibility, 412 did not meet the inclusion criteria and 133 patients declined to participate. (Fig 1).

### Randomization and intervention

Prior to enrollment the treatment assignments were generated as a block random sample, with 50 patients assigned to each treatment arm in blocks of 100 patients. The randomization was carried out in R version 3.1.1 (Vienna, Austria). The study statistician provided the principal investigator with a list of 400 random assignments of potential study subjects that were randomized in blocks of 100 (50 for each study arm within each block of 100.) Patients meeting eligibility criteria were numbered chronologically based on the date of consent and were assigned to each arm according to the randomization list. All patients in the tested group received a physician order for pharmacogenetic testing. Patients were enrolled from February 2015 to December 2015 and each patient was followed for 60 days from date of hospital discharge with follow-up ending in February 2016. The study enrolled a total of 110 patients comprised of 53 controls and 57 interventional recipients.

Baseline data was extracted from patient charts and manually entered into the study database. The study pharmacist reviewed drug-drug interactions (DDIs), DGIs, and cumulative drug and/or gene interactions in the tested group using the YouScript^®^ generated reports and CDST to provide drug therapy recommendations to clinicians, after which prescriptions were altered at clinician discretion. Patients in the untested group were screened for DDIs using standard drug information resources (Lexicomp) with recommendations acted on according to clinician judgment. Time to change therapy was similar to those in the tested group.

### Outcome measures

The primary outcomes included the number of re-hospitalizations and ED visits at 30 and at 60 days. The exploratory outcomes included the time to the first re-hospitalization, time to the first ED visit, number of falls during the study, deaths, scores on select Outcome and Assessment Information Set (OASIS) quality metrics at 30 and 60 days, and clinical decision-making (proportion of recommendations accepted by study pharmacist and passed on to clinicians and the proportion of recommendations accepted by clinicians). The selected OASIS metrics included overall status, pain, confusion, anxiety, disruptive behavior, assistance with activities of daily living, and depression. Data was extracted from the patient chart upon admission and recorded using a case report form (CRF) at 30 days and 60 days. All data was manually entered into the study database.

### Genotyping and CDST

Buccal samples were obtained from eligible patients for determination of genotype and shipped to Genelex Corporation (Seattle, WA). Genelex is accredited by the College of American Pathologists (CAP 1073709); certified under the Clinical Laboratory Improvement Amendments (CLIA No. 50D0980559); is Washington State Medical Test Site No. MTS-60353885; New York State Department of Health license no. PFI 8201; and licensed to perform high complexity clinical testing in all US states. DNA extractions from buccal swabs were performed using the MagJET genomic DNA extraction kit from Thermo Fisher (Waltham, MA). Genotypes were obtained using a laboratory-developed, multiplex PCR based tests followed by single base primer extension for variant detection by mass spectrometry (MassArray Analyzer 4 System, Agena Bioscience, San Diego, CA). Pharmacogenetic testing included PCR based assays to detect all common and rare variants with known clinical significance at analytical sensitivity and specificity greater than 99% [18]. The tested alleles included *CYP2C9*: *2,*3,*4,*5,*6,*8,*11,*13,*15; *CYP2C19*: *2,*3,*4,*5,*6,*7,*8,*9,*10,*12,*17; *CYP2D6*: *2,*2A,*3,*4,*5,*6,*7,*8,*9,*10,*11,*12,*14, *15, *17,*19,*20,*29,*35,*36,*41, gene deletion and duplications; *CYP3A4*: *22; *CYP3A5*: *3; and *VKORC1*: c.-1639G>A. The gene panel was decided upon based on the high frequency of variation and the variety of common medications that it effects. The CYPs selected are the CYPs that have been shown to have a consistent relationship with drug levels [4, 5, 19, 20]. The absence of a positive test result for all variants listed results in the assignment of a *1 wild type status.

The pharmacogenetic definition (*CYP2C9*, *CYP2C19*, *CYP2D6*, *CYP3A4* and *CYP3A5*) and inferred phenotypes calling (ultrarapid, normal, intermediate and poor metabolizer) is consistent with the recently published guidance for allele function status and phenotype [21]. Two increased function alleles, or more than two normal function alleles were inferred as ultrarapid metabolizer; combinations of normal function and decreased function alleles were inferred as normal metabolizer; combinations of normal function, decreased function, and/or no function alleles were inferred as intermediate metabolizer, and combinations of no function alleles were inferred as poor metabolizer. In addition, the combinations of *CYP2C19* normal function and increased function allele was inferred as ultrarapid metabolizer; and the combinations of *CYP2C19* increased and no function allele were inferred as intermediate metabolizer based on *CYP2C19* published guidelines [22]. The *CYP3A5* phenotype designations, non-expressers (poor metabolizer), intermediate expressers (intermediate metabolizer) and expressers (normal metabolizer) are based on the *CYP3A5* guidelines [23]. With respect to *VKORC1*, a common noncoding variant (*c*.*-1639G>A*, rs9923231) is significantly associated with warfarin sensitivity and reduced dose requirements, as *c*.*-1639A* carriers require lower initial warfarin doses than *c*.*-1639G* carriers [24].

Patient phenotypes and medication lists were analyzed by YouScript^®^ and verified by a clinical pharmacist. YouScript^®^ is a CDST (S1 Appendix) that performs a comprehensive analysis of patient medication regimen and their genetics using a proprietary algorithm and a curated database of the primary literature to predict changes in drug levels [15, 16]. The report (S1 Appendix) included type and date of sample collection, the tests conducted, the genotype for each gene tested, the patient’s genetic phenotype for each gene tested, the medications the patient was taking, and prescribing suggestions. YouScript^®^ also categorizes the type of interaction (DDI, DGI, and cumulative drug and/or gene interactions) and assigns a warning category. The strongest warning is “contraindicated” followed by “major”, “moderate” and “minor” impact. Evidence-based medication or dose change guidance was provided for all “contraindicated”, “major” and “moderate “warnings. Reports were reviewed by the study pharmacist who provided final drug and dose change recommendations to fit patient needs and forwarded to clinicians.

### Statistical analysis

Statistical analysis compared the primary and exploratory outcomes between tested and untested groups. Descriptive statistics are presented as means and ranges for count and ordinal outcomes and as percentages for binary outcomes. All count outcomes were compared between tested and untested groups using Poisson regression, and all Poisson regression models were diagnosed for over-dispersion. When over-dispersion was detected (p<0.05), the negative binomial regression was used instead of the Poisson regression. substantial The log-rank test was used to compare time-to-event outcomes. The time-to-event outcomes were further described using Kaplan-Meier estimates (curves and estimates at 30 and 60 days shown) and hazard ratios estimated from the Cox proportional hazards model. Ordinal outcomes (OASIS metrics and PHQ-2 scores) were compared using the Wilcoxon rank sum test. Fisher’s exact test was used to compare the proportion of patient deaths. The effect on mortality was expressed by a risk ratio calculated from a log-link generalized linear model (with confidence interval calculated using profile likelihood from the model). Patient deaths and the composite event outcomes (analyzed both as counts and times to the first event) were added post-hoc to the exploratory outcomes. Composite event outcomes included re-hospitalizations + ED visits + deaths and re-hospitalizations + ED visits (where overlapping events were counted as one). Both were compared at 30 and at 60 days and as the time to the first composite outcome event. Calculations were carried out in R, version 3.2.3 (Vienna, Austria). All tests were two-sided. P<0.05 was used to denote statistical significance. As the study was designed as a pilot, tests were not adjusted for multiple comparisons.

## Results

A total of 57 tested patients were compared to 53 untested patients (Fig 1). Table 1 reports patient demographics overall and for tested and untested groups. The mean (n = 110) age was 75.6 years and 81.8% of the patients were 65 years and older. The percentage of female patients in the tested group and untested group were 56.1% and 67.9% respectively. The population in the area where the trial was conducted is predominately white—86.8% in 2010 [25]. The average pharmacogenetic risk, the likelihood that testing would reveal substantial gene based drug interactions, for the tested and untested groups was 33.2% and 34.3% respectively. The mean OASIS score for overall health at the start of intervention in the tested and untested groups was 2.64 and 2.63 respectively (S1 Table).

**Table 1:** Demographic characteristics of patients.

Demographics		Overall (n = 110)		Tested (n = 57)		Untested (n = 53)	
		n	%	n	%	n	%
**Gender**	Male	42	38.2	25	43.9	17	32.1
	Female	68	61.8	32	56.1	36	67.9
**Race**	White	109	99.1	57	100	52	98.1
	African American	1	0.9	0	0	1	1.9
**Age**	50–64 yrs	20	18.2	6	10.5	14	26.4
	65+ yrs	90	81.8	51	89.5	39	73.6
**Overall Age**		**Mean**	**SD**	**Mean**	**SD**	**Mean**	**SD**
		75.6	10.7	76.5	9.4	74.6	11.9

%, percentage; n, number of patients, SD, standard deviation; yrs, years.

Tables 2 and 3 compare primary and exploratory composite outcomes between the tested group and the untested group at 30 and 60 days post-discharge. Reduction in the number of re-hospitalizations, ED visits and in the composite number of outcomes were observed at 30 days. At 60 days, primary and exploratory composite outcomes post-discharge were considerably lower in the tested group. As shown in Table 2, at 60 days twenty-eight patients in the untested group had no ED visit, while eighteen had one visit, five had two visits, one had three visits and one had four visits. In the tested group thirty-nine patients had no ED visits at 60 days while fourteen had one visit and four had two visits. At 60 days thirty-one patients in the untested group had no re-hospitalization, while thirteen had one re-hospitalization, three had two re-hospitalizations and six had three re-hospitalizations. In the tested group forty-one patients had no re-hospitalization at 60 days while fourteen had one re-hospitalization, one had two re-hospitalizations and one had three re-hospitalizations. As shown in Table 3, the mean number of outcomes per patient in the tested vs. untested group for re-hospitalizations was 0.33 vs. 0.70 (relative risk (RR), 0.48; 95% confidence interval (CI), 0.27–0.82; p = 0.007); ED visits was 0.39 vs. 0.66 (RR, 0.58; 95% CI, 0.34–0.99; p = 0.045); re-hospitalizations + ED visits was 0.54 vs. 1.04 (RR, 0.52; 95% C, 0.32–0.86; p = 0.01). The testing reduced the number of re-hospitalizations, ED visits, and composite number of re-hospitalization + ED visits at 60 days by 52%, 42% and 48% respectively. The true re-hospitalization, ED visits, and composite number of re-hospitalization + ED visits reduction in a population similar to this study will likely fall between 18% to 73%, 1% to 66%, and 14% to 68% (95% CI) respectively. Six deaths were observed in the untested group versus one death in the tested group, an 85% reduction in the risk of death in the tested group (RR, 0.15; 95% CI, 0.01–0.87; p = 0.054). At 60 days, the mean composite number of re-hospitalizations + ED visits + deaths was 0.54 for the tested group versus 1.10 for the untested group (RR, 0.50; 95% CI, 0.30–0.81; p = 0.005). For the composite exploratory outcomes at 60 days, the dispersion assumption was violated, so negative binomial regression was used instead. At 30 days, tested group events were lower but the effect was smaller than at 60 days.

**Table 2:** The number of patients by the number of events for each primary outcome, by the treatment group.

Events	0 events	1 event	2 events	3 events	4 events
**Number of Re-hospitalizations (30 days)**					
Untested	38	10	5	0	0
Tested	46	9	1	1	0
**Number of Re-hospitalizations (60 days)**					
Untested	31	13	3	6	0
Tested	41	14	1	1	0
**Number of ED Visits (30 days)**					
Untested	36	13	4	0	0
Tested	44	12	1	0	0
**Number of ED Visits (60 days)**					
Untested	28	18	5	1	1
Tested	39	14	4	0	0

ED, emergency department; Untested group (n = 53); Tested group (n = 57).

**Table 3:** Comparison of primary and exploratory outcomes between the treatment groups.

Outcomes	Untested (n = 53)	Tested (n = 57)	RR (95% CI)*	p value*
	Mean (range)	Mean (range)		
**Number of Re-hospitalizations** (Primary Outcome)				
At 30 days	0.38 (0–2)	0.25 (0–3)	0.65 (0.32–1.28)	0.21
At 60 days	0.70 (0–3)	0.33 (0–3)	0.48 (0.27–0.82)	0.007
**Number of ED visits** (Primary Outcome)				
At 30 days	0.40 (0–2)	0.25 (0–2)	0.62 (0.31–1.21)	0.16
At 60 days	0.66 (0–4)	0.39 (0–2)	0.58 (0.34–0.99)	0.045
**Composite Number of Re-hospitalizations + ED visits** (Exploratory Outcome)				
At 30 days	0.57 (0–2)	0.37 (0–3)	0.65 (0.37–1.13)	0.13
At 60 days	1.04 (0–4)	0.54 (0–3)	0.52 (0.32–0.86)	0.01**
**Composite Number of Re-hospitalizations + ED visits + Deaths** (Exploratory Outcome)				
At 30 days	0.58 (0–2)	0.37 (0–3)	0.63 (0.36–1.09)	0.10
At 60 days	1.10 (0–5)	0.54 (0–3)	0.50 (0.30–0.81)	0.005**

CI, confidence interval; ED, emergency department; RR, relative risk;

*Poisson regression model;

** the dispersion assumption violated and the negative binomial regression was used instead.

**Fig 2:**
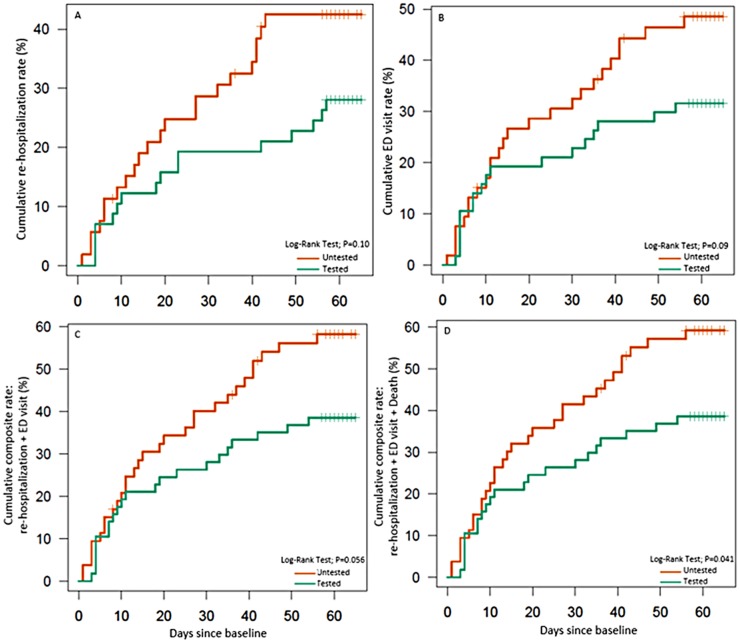
Cumulative rate (%) for re-hospitalizations, ED visits, and composite events using Kaplan-Meier estimator. 2A and 2B represent the Kaplan-Meier curves for the time to first re-hospitalization and to the first ED visits, respectively. 2C and 2D represent the Kaplan-Meier curves for the time-to-cumulative composite event outcomes for re-hospitalizations + ED visits and re-hospitalizations + ED visits + deaths, respectively. ED, emergency department; p, p value. The vertical lines on the survival curves show the times when event-free subjects reach the end of their protocol pre-specified follow-up of approximately 60 days (55–65 days).

Table 4 compares the exploratory time-to-event outcomes between the tested and untested groups at 30 days and 60 days. The hazard ratio (HR) was 0.59 for time to the first re-hospitalization and 0.60 for time to the first ED visit. The HR for the time-to-composite event outcomes for re-hospitalizations + ED visits and re-hospitalizations + ED visits + deaths was 0.59 (95% CI, 0.34–1.02; P = 0.056) and 0.57 (95% CI, 0.33–0.99; P = 0.041), respectively. Fig 2A and 2B presents the Kaplan-Meier curves for the time to first re-hospitalization and to the first ED visits. At 60-days, the cumulative rate in the tested versus untested group for re-hospitalization rate was 28% vs. 43% (log-rank test for the entire follow-up: P = 0.10); cumulative ED visit rate was 32% vs. 49% (P = 0.09). Fig 2C and 2D presents the Kaplan-Meier curves for the time-to-cumulative composite event outcomes for re-hospitalizations + ED visits and re-hospitalizations + ED visits + deaths.

**Table 4:** Comparison of the time-to-event outcomes between the untested and tested groups.

Time-to-event Outcomes	Cumulative % events at 30 days		Cumulative % events at 60 days		p value*	HR (95% CI)
	Untested	Tested	Untested	Tested		
Days to the first Re-hospitalization	29%	19%	43%	28%	0.10	0.59 (0.31–1.12)
Days to the first ED visit	30%	23%	49%	32%	0.09	0.60 (0.33–1.10)
Composite event: Re-hospitalization + ED visit	40%	28%	58%	39%	0.056	0.59 (0.34–1.02)
Composite event: Re-hospitalization + ED visit + Death	42%	28%	59%	39%	0.041	0.57 (0.33–0.99)

ED, emergency department; HR, hazard ratio values at 60 days, Cox proportional hazard model; CI, confidence interval;

*p-value (log-rank test);

%, percentage; cumulative % events were estimated by the Kaplan-Meier estimator.

The comparison of selected OASIS metrics between the tested and untested group is addressed in S1 Table. Scores were evaluated and documented at time of admission to home health, at 30 days, and at 60 days following enrollment for improvement in overall status, pain, confusion, anxiety, depression, disruptive behavior, and the need for assistance with activities of daily living. The differences in the overall all scores at 30 days and 60 days between the tested and untested groups were relatively small. The mean number of falls per patient was 0.11 and 0.09 for the tested and untested groups respectively.

Tables 5 and 6 compare differences in the distribution of CYP 450 metabolic phenotypes, and differences in the severity of DDI (binary and cumulative), DGI, or DDGI between this home health trial and another published study population [16]. As seen in Table 5, for each of the genes of interest a large fraction of patients were characterized as having a “risk phenotype”. The phenotype breakdown of evaluated patients, by enzyme, was as follows: 42.9% were intermediate or poor *CYP2D6* metabolizers; 64.9% were intermediate, poor, or ultra-rapid *CYP2C19* metabolizers; 29.8% were intermediate or poor *CYP2C9* metabolizers; 10.5% intermediate *CYP3A4* metabolizers; and 12.3% were *CYP3A5* intermediate expressers. The mean number of all YouScript^®^ recommendations per patient in the tested group was 2.18 and the mean number of gene-based recommendations was 1.49. The percentage of tested patients showing overall YouScript^®^ interaction severity with “change or major” recommendations was 35.1% and with “consider or moderate” recommendations was 21.1% (Table 6). YouScript^®^ recommendations were accepted by the study pharmacist who reviewed the recommendations based on additional patient factors and needs and then provided final recommendations to clinicians. Of the 124 recommendations passed on to clinicians, 96 were followed (77%), 6 were not followed (5%), and 22 were unknown (18%). By unknown, it meant that it could not be determined whether the reviewing physician followed a specific prescribing suggestion.

**Table 5:** Distribution of evaluated cytochrome P-450 (CYP) metabolic phenotypes between the Home Health Trial and another published study population [1,6].

Phenotype	Hocum et al. Prevalence, %	Home Health Trial Prevalence, %
***CYP2D6***^#^		
Normal Metabolizer	52.8	57.1
Intermediate Metabolizer	37.7	37.5
Poor Metabolizer	6.8	5.4
Ultrarapid Metabolizer	2.7	0.0
***CYP2C19***		
Normal Metabolizer	42.1	35.1
Intermediate Metabolizer	25.8	31.6
Poor Metabolizer	2.6	1.8
Ultrarapid Metabolizer	29.5	31.5
***CYP2C9***		
Normal Metabolizer	67.5	70.2
Intermediate Metabolizer	29.1	26.3
Poor Metabolizer	3.4	3.5
***CYP3A4***		
Normal Metabolizer	92.4	89.5
Intermediate Metabolizer	7.6	10.5
***CYP3A5***		
Non-Expresser	72.3	87.7
Intermediate Expresser	20.5	12.3
Expresser	7.2	0.0

^**#**^When gene duplication is detected for *CYP2D6* alleles of different activity (e.g. one inactive allele and one active allele), the technology cannot distinguish which allele is duplicated and therefore cannot assign a definitive phenotype. There were 560 *CYP2D6* phenotype results in the AJHP cohort and 1 result in the Home Health Trial cohort affected by this, and they were omitted from this phenotype prevalence analysis.

**Table 6:** Comparison of YouScript^®^ recommendation severity of drug-drug, drug-gene, and drug-drug-gene interaction between the Home Health Trial and another published study population [16].

Interaction Severity	Hocum et al.(n = 20534), %	Home Health Trial (n = 57), %
**Overall**^a^		
Change/Major	8.9	35.1
Consider/Moderate	36.7	21.1
Monitor/Minor	23.5	31.5
No Change/None	30.9	12.3
**DDI**^b^		
Change/Major	5.4	22.8
Consider/Moderate	22.9	19.3
Monitor/Minor	8.3	7.0
No Change/None	63.5	50.9
**DGI/DDGI**^c^		
Change/Major	3.9	17.5
Consider/Moderate	23.4	28.1
Monitor/Minor	28.4	29.8
No Change/None	44.3	24.6

^a^ Most severe drug-drug, drug-gene, or drug-drug-gene interaction detected for each patient

^b^ Most severe drug-drug (binary and cumulative) interaction detected for each patient

^c^ Most severe drug-gene or drug-drug-gene interaction detected for each patient

## Discussion

Pharmacogenetic testing of genes coding for drug metabolizing enzymes has the potential to optimize medication prescribing, dosing, and monitoring by identifying metabolizer phenotype status. Although over 137 medications have pharmacogenetic considerations in their label [20], the value of testing has yet to be established. Although some validation is needed, it should be noted that approximately one-half of the most commonly prescribed drugs in the United States undergo metabolism via CYP450 enzymes. Similar to creatinine clearance (CrCl) rate as a biomarker of kidney function, CYP genetic status serves as a biomarker of the patient’s ability to metabolize drugs via CYP450 enzymatic pathways. FDA guidance regarding pharmacokinetics in patients with impaired renal function suggests that dosing recommendations be based on creatinine clearance or estimated glomerular filtration rate (eGFR) and other relevant pharmacokinetic parameters, such as area under the curve (AUC), half-life and C_max_ [26]. Similarly, patient CYP genetic status and the resulting alterations to CYP enzyme activity are fundamental to understanding the AUC, half-life and C_max_ which measure and determine circulating drug levels. Older patients may realize a greater benefit from pharmacogenetic testing along with an appropriate CDST being at heightened risk for polypharmacy and adverse medication outcomes [27]. This trial assessed the clinical impact of pharmacist medication management with access to pharmacogenetic profiling integrated with cumulative drug-gene interaction warnings (YouScript^®^ CDST). Inhibition of CYP enzymatic activity by concomitant medication(s) can dramatically change a person’s metabolic capacity presenting with a phenotype that does not match with the genetic phenotype [28]. YouScript^®^ algorithms combine all drug-gene and drug-drug interactions to predict the cumulative effect of all interacting components on an affected drug. This is important because normal and intermediate metabolizers can be ‘phenoconverted’ to poor metabolizers. The phenotype prediction from genotype data depends heavily on the allelic variants present in a population of interest and the number of alleles being interrogated. An overestimate of the normal-function of CYPs is likely to occur when a considerable number of no- and decreased-function alleles are missed. In addition, other genetic, physiological, pathological, and environmental factors on CYP expression and activity may also have an implication. Interpretation of the functional consequences of predicted phenotypes for drug clearance *in vivo*, and thus translation into specific dosing guidelines for individual drug-diplotype pairs, will benefit from future genotype-stratified pharmacokinetic studies for high-priority drugs [29].

The study population was selected to have a higher than average return on pharmacogenetic testing defined by the likely frequency of drugs interacting with CYPs. Patients who were aged 50 and older, enrolled in home health, and taking or initiating treatment with at least one medication with a significant DDI or DGI as defined by FDA boxed warning, FDA cautionary labeling, clinical literature or YouScript^®^ algorithm- predicted significant effect were included. To verify patients were similar in each arm, the distribution of diagnosis (patient diseases, disorders and related health problems) and medication utilization between the tested and untested groups were compared. The diagnosis results (S2 Table) showed significant differences only in the frequency of the cerebrovascular conditions and eye diseases. Finding statistically significant differences for two conditions is not unusual among twenty-six comparisons (1.3 such differences would be expected, on average.) Additionally, the frequencies of these two conditions were higher in the tested group. Despite this, there was an overall reduction in ED visits and re-hospitalizations at 60 days. Therefore, not adjusting for this imbalance appears to be, in fact, conservative. All other diseases, disorders and health related problems between the two groups were not statistically significant and likely did not impact the presented outcomes of the study.

Home health patients are polypharmacy treated subjects; the average number of drugs per patient in this study was 11.6. The average drug count at baseline (before pharmacogenetic profiling based intervention) was relatively balanced between the tested and untested groups (11.6 vs. 11.8; p = 0.6, two sample t-test). On comparing the frequency of drugs prescribed between the two groups (S3 Table), no major difference were observed validating a relatively good balance between the two arms. A major 52% reduction in the number of re-hospitalizations and a 42% reduction in the number of ED visits at 60 days post-discharge was observed in the tested versus the untested group. This finding occurred in an HHA that, according to 2015 Home Health Quality Improvement reporting was in the 5^th^-12^th^-percentile group for the CMS re-hospitalization benchmark. The HHA received 4.5 of 5 stars in the CMS Home Health Compare rating.

Reducing rates of re-hospitalization has attracted attention from policymakers as a way to improve quality of care and reduce costs. ED visits are also an important cause of morbidity, and may often be caused by ADEs, particularly among patients aged 65 years and older [30]. Six deaths were observed in the untested group versus one death in the tested group, an 85% reduction in the risk of death. However, because of the small sample size it is unclear if it was directly related to pharmacogenetic-guided treatment. The death in the tested group occurred before pharmacogenetic results were sent to the clinician.

The fact that most patients are not normal metabolizers for at least one CYP drug metabolizing enzyme is extremely important when managing a patient medications. The wide range of CYP isozyme-encoding gene variants suggests that a large number of DGIs and DDGIs must go undetected without pharmacogenetic testing. The total number of patients with risk phenotypes, as well as the incidence of DGIs and DDGIs identified, supports the use of risk-based pharmacogenetic testing [16]. Interestingly, this study had many more severe interactions (Table 6) than the Hocum et al study [16] likely because of higher average drug count (11.6 vs. 8.2). In addition, the mean age, percentage of patients who were 65+ years, and the racial differences were higher in this study (S4 Table). Also, the home health patients in this study are all being released from the hospital to home health, so they tend to be less well than a typical ambulatory patient.

The limitations of this study include small sample size, use of a randomized population within one institution and undetermined impact of the genetic testing on patient-provider interactions. Also, the average time to change drug therapy was approximately 3 weeks; only one week short of the 30-day time point which could have impacted the time-to-event outcome at 30 days. However, the benefits of pharmacogenetic profiling at 30 days are possible if the average time to change therapy is shortened, but the impact is still less certain since in some instances as it may take longer for the benefits of the intervention to materialize. This study was planned as a pilot study and therefore no power calculations were carried out. Despite the small size of the study, other factors such as age, gender, race and baseline health status (OASIS) were relatively balanced between the two study arms. Because of this balance, the small size of the study and the type of the outcomes, the analyses were not adjusted for covariates to avoid an increased type I error [31, 32]. When results were adjusted for categorical age all p-values were smaller than the corresponding unadjusted p-values. The unadjusted p-values presented in our results are thus appropriately more conservative compared to the adjusted p-values. Actual cost and charge data was not available. Therefore, cost savings were modeled based on Medicare average all-cause readmission [33] and ED visit cost [34], resulting in a $4382 per patient cost savings in 60 days (S5 Table) prior to the cost of intervention, resulting in decreased healthcare resource utilization. Follow-up was limited to 60 days post-discharge, but over time additional savings could increase the value obtained from the one-time expense of testing as the results can be used for ongoing patient management. A recently conducted cost-effectiveness analysis showed cost-savings when considering a one-time genetic test to avoid lifetime ADRs [35].

In conclusion, we conducted a pragmatic, prospective, randomized controlled clinical trial testing comprehensive medication management delivered by clinical pharmacists, incorporating pharmacogenetic testing along with appropriate CDST versus usual care. This home health trial mirrored a previously published prospective observational study which demonstrated that pharmacogenetic CYP testing of ambulatory elderly exposed to polypharmacy reduced hospitalizations by 39% and ED visits by 71% compared to a matched retrospective control cohort. This study offers a potentially important opportunity to minimize ADEs and reduce health resource utilization and marks an important step towards accomplishing the goals of the Triple Aim (improved care of the individual, improved health of the patient population and decreased per capital costs) [36]. To further validate these findings, additional prospective confirmatory studies involving real-world applications in larger populations in the same and alternate settings are needed to broaden its acceptance in routine clinical practice.

## Supporting information

S1 Data Dataset used for the analysis. (XLSX)

S1 Checklist CONSORT checklist. (DOC)

S1 Protocol Trial protocol and statistical analysis plan. (PDF)

S1 Text Institutional Review Board approval from Harding University. Also includes amendment submission and approval from IRB.
(PDF)

S1 Appendix Description of the YouScript^®^ CDST and example of the personalized prescribing report. (PDF)

S1 Table Comparison of selected OASIS and PHQ-2 metrics between the two groups. (DOCX)

S2 Table Comparison of patient diseases, disorders and related health problems between the two groups. (DOCX)

S3 Table Comparison of the frequency of drugs prescribed between the two groups. (DOCX)

S4 Table Comparison of differences in the demographic characteristics of patients between this trial and Hocum study. (DOCX)

S5 Table Estimated financial savings from re-hospitalizations and ED visits reduction. (DOCX)

## Abbreviations

ADE: adverse drug event
ADR: adverse drug reaction
CDST: clinical decision support tool
CI: confidence interval
CRF: case report form
DDGI: drug-drug-gene Interactions
DDI: drug-drug interactions
DGI: drug-gene interactions
ED: emergency department
HHA: home health agencies
HR: hazard ratio
OASIS: Outcome and Assessment Information Set
RR: relative risk
