# Supplementary material for: Clinical impact of pharmacogenetic profiling with a clinical decision support tool in polypharmacy home health patients: A prospective pilot randomized controlled trial
Source: PLoS One. 2017 Feb 2;12(2):e0170905. doi: 10.1371/journal.pone.0170905 (PMC5289536; doi:10.1371/journal.pone.0170905)
Supplement: S1 Protocol — (PDF) [file pone.0170905.s003.pdf]

Clinical Trial Protocol

A Pilot Prospective, Randomized Controlled Trial Assessing the Clinical Impact of Integrated Pharmacogenetic Testing on Selected OASIS Metrics, Re-hospitalizations and Emergency Department visits

Principal Investigator: Lindsay Elliott, Pharm.D., CGP
Harding University
915 E. Market Ave, Box 12230
Searcy, AR 72149-5615
(865) 591-2533
lselliott@harding.edu

Clinical site: White County Medical Center Home Health
3109 East Moore St,
Searcy, AR 72143
Lindsay Elliott, Pharm.D., CGP
(865) 591-2533
lselliott@harding.edu

Lead study nurse: Vicki Pennington
3109 Ease Moore St,
Searcy AR 72143
vpennington@wcmc.org

Laboratory: Genelex Corporation
3101 Western Ave
Seattle, WA 98121
1.800.523.3080

Protocol version number: 1.0

December 16, 2014

TABLE OF CONTENTS

1 BACKGROUND & RATIONALE 7
 1.1 Overview 7
 1.2 Rationale 10
 1.3 Hypotheses 10
2 STUDY DESIGN & OBJECTIVES 10
 2.1 Primary objective 11
 2.2 Exploratory: 11
3 STUDY PROCEDURES 11
 3.1 Inclusion Criteria 11
 3.2 Exclusion Criteria 12
 3.3 Recruitment plan 13
 3.4 Randomization Procedures 13
 3.5 Retention Plan 15
 3.6 Subject Withdrawal 15
4 ETHICAL CONSIDERATIONS 15
 4.1 Statement of compliance 15
 4.2 Risks 15
 4.3 Benefits 16
 4.4 Informed Consent Process 16
 4.5 Subject Confidentiality 16
 4.6 Institutional Review Board 16
 4.7 Reporting Unanticipated Problems to the IRB 16
 4.8 Unanticipated Problem Reporting to IRB 17
5 DATA MANAGEMENT CONSIDERATIONS 17
 5.1 Outcome measures 17
 5.2 Source documents and access to source data/documents 19
 5.3 Quality control and quality assurance 19
6 DATA CONSIDERATIONS 19
7 STATISTICAL CONSIDERATIONS 20
8 RESULTS DISSEMINATION 20
9 APPENDICES 20

LIST OF ABBREVIATIONS

ADE	Adverse Drug Event
ADL	Activities of Daily Living
ADR	Adverse Drug Reaction
AE	Adverse Event/Adverse Experience
CFR	Code of Federal Regulations
CRF	Case Report Form
GCP	Good Clinical Practice
ICF	Informed Consent Form
ICH	International Conference on Harmonisation
IRB	Institutional Review Board
N	Number (typically refers to subjects)
OHRP	Office for Human Research Protections
PHI	Protected Health Information
PI	Principal Investigator
QA	Quality Assurance
QC	Quality Control
SOP	Standard Operating Procedure
UP	Unanticipated Problem

PROTOCOL SUMMARY

Title: A Pilot Prospective, Randomized Controlled Trial Assessing the Clinical Impact of Integrated Pharmacogenetic Testing on Selected OASIS Metrics, Re-hospitalizations and Emergency Department visits

Synopsis: Patients meeting eligibility criteria will be randomized into two groups, one receiving pharmacogenetic testing and the other not receiving pharmacogenetic testing. In this open-label trial, a pharmacist will make medication therapy recommendations using YouScript® Personalized Prescribing System for patients who receive genetic testing and standard drug information resources per usual for patients who do not undergo pharmacogenetic testing.

Both groups will be followed for 60 days. The number of re-hospitalizations and emergency department (ED) visits will be recorded as well as time to first re-hospitalization and time to first ED visit. Select OASIS metrics (e.g. M1034, M1242, M1710, M1720, M1745, M2110) and depression using PHQ-2 will be evaluated and documented at time of admission to home health, at 30 days, and at 60 days for improvement in overall status, pain, confusion, anxiety, depression, disruptive behavior, and the need for assistance with activities of daily living (ADLs) and instrumental activities of daily living (IADLs). The number of falls will be collected as well as the proportion of YouScript® recommendations accepted by study pharmacist and passed on to clinicians and the proportion of recommendations accepted by clinicians.

Objectives: Primary:

- To assess the number of re-hospitalizations at 30 and 60 days post discharge with pharmacogenetic testing and YouScript® Personalized Prescribing System
- To assess the number of Emergency Department visits at 30 and 60 days post discharge with pharmacogenetic testing and YouScript® Personalized Prescribing system

Exploratory:

- To assess time to first re-hospitalization.
- To assess time to first Emergency Department visit.
- To assess the impact of genetic testing on overall status according to OASIS M1034 at 30 and 60 days post discharge.
- To assess the impact of genetic testing on frequency of pain according to OASIS M1242 at 30 and 60 days post discharge.
- To assess the impact of genetic testing on frequency of confusion according to OASIS M1710 at 30 and 60 days post discharge.
- To assess the impact of genetic testing on frequency of anxiety

according to OASIS M1720 at 30 and 60 days post discharge.

- To assess the impact of genetic testing on depression according to PHQ-2 at 30 and 60 days post discharge.
- To assess the impact of genetic testing on frequency of disruptive behavior according to OASIS M1745 at 30 and 60 days post discharge.
- To assess the impact of genetic testing on the frequency of ADL and IADL assistance according to OASIS M2110 at 30 and 60 days post discharge.
- To assess whether YouScript® testing decreases falls
- To assess the proportion of YouScript® Personalized Prescribing System recommendations accepted by the study pharmacist and passed on to clinicians.
- To assess the proportion of study pharmacist recommendations acted on by clinicians.

**Sample size**	100 patients (50 intervention, 50 controls)
**Population:**	The study population consists of patients aged 65 and older who are currently enrolled in home health and are presently taking or initiating treatment with at least one oral form of medication with a significant drug-drug or drug-gene interaction as defined by FDA boxed warning, FDA cautionary labeling, clinical literature or a YouScript® algorithm-predicted significant effect.<br><br>Those meeting eligibility criteria will be prospectively enrolled in either the “tested” group and undergo YouScript® testing or “untested” group and not undergo YouScript® testing.
**Description of Intervention:**	Patients in the “tested” group will receive genetic testing. The study pharmacist will review drug-drug, drug-gene, and drug-drug-gene interactions using YouScript® Personalized Prescribing System to provide drug therapy recommendations to prescribers.
**Estimated Time to Complete Enrollment:**	Up to 20 weeks (5+ months)
**Subject Participation Duration:**	Each patient will be followed for 60 days from date of admission to home health
**Total Study Duration:**	Up to 8 months, depending on the rate of accrual

Figure 1: Schematic of Study Design.

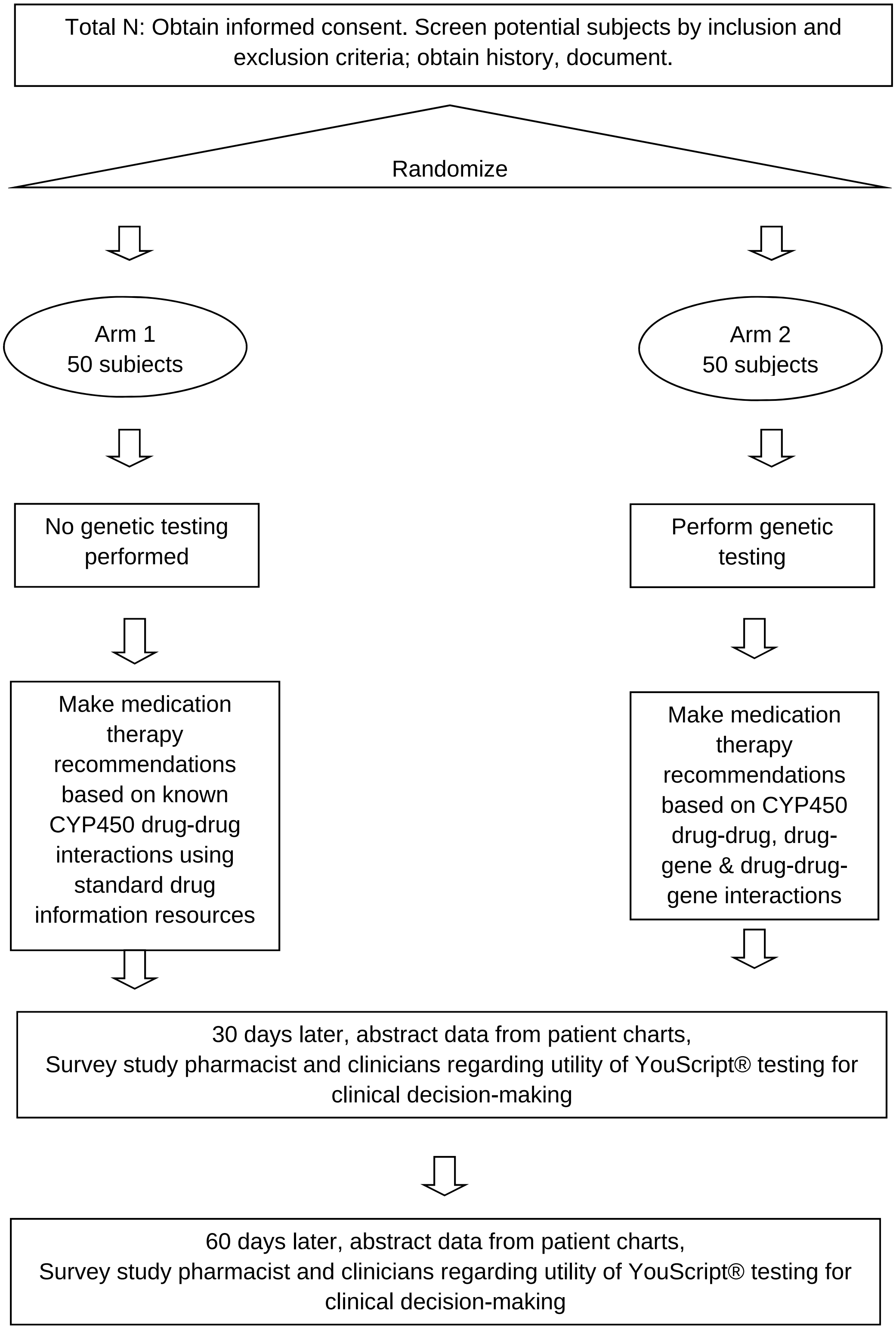

1 BACKGROUND & RATIONALE

1.1 Overview

Drug-drug interactions (DDIs) are widely recognized as a major cause of adverse drug reactions. Numerous drug databases are available for pharmacists and clinicians to check for potential DDIs. More recently discovered and vitally important are drug-gene interactions (DGIs) and drug-drug-gene interactions (DDGIs). Cytochrome P-450 enzymes reduce or alter the pharmacologic activity of many drugs and facilitate their elimination. Additionally, genetic variance among patients who are abnormal metabolizers compounds the potential risk for adverse effects and often results in decreased efficacy. Variance in drug levels presents a major therapeutic problem in dosage optimization. [1]

At this point, clinicians can only determine potential DDIs but not DGIs and DDGIs. Knowledge of genetic variations in metabolism within a patient can potentially circumvent adverse effects and allow proper dosage adjustments. YouScript® Personalized Prescribing System, by including a patient's genetic profile, uses an algorithm to flag actual and potential DDIs, DGIs, and DDGIs, provides a list of safer alternatives, contains package inserts for medications, and has direct links to evidence-based literature to support recommendations. Having access to such a database can allow for "personalized prescribing" resulting in decreased hospitalizations, medication-related adverse effects, and emergency department visits while increasing patient efficacy and quality of life and improving clinical-decision making.

1.1.1 Home health patients

The majority of patients receiving home health care are aged 65 and older, take multiple medications, and have poor compliance due to adverse drug effects, which often times leads to emergency department visits, hospitalizations, and decreased quality of life. Knowledge of a patient's genetic profile will allow clinicians to make medication therapy decisions specific to each patient leading to better outcomes such as decreased pill burden, improvement of disease state, decreased adverse drug reactions, and increased quality of life. Better outcomes will reduce medical costs for both patients and healthcare systems.

1.1.2 Medication-Related Problems

Medication-Related Problems (MRPs) are common among people who take multiple medications. MRPs often result in Adverse Drug Reactions (ADRs) or a therapeutic failure. A medication-related problem (MRP) is as an event or circumstance
involving drug treatment that actually or potentially interferes with the patient experiencing an optimum outcome of medical care.[2] Classifications of MRPs include poor compliance, adverse drug reactions, drug interactions, unnecessary drug therapy, additional medication therapy need, and dosage too high/low, etc. Patients are harmed by MRPs. For example, routinely prescribed psychiatric medications are a common cause of adverse drug reaction

---

[1] Wilkinson GR. Drug Metabolism and Variability among Patients in Drug Response. NEJM 2005; 352(21): 2211-21.
[2] Hepler CD, Strand LM. Opportunities and responsibilities in pharmaceutical care. Am J Hosp Pharm.1990;47:533-43.

driven emergency room visits[3]. Emergency room visits and hospitalizations associated with drug therapy problems undoubtedly increase healthcare system costs[4].

1.1.3 OASIS metrics

The Centers for Medicare and Medicaid Services (CMS) requires that Home Health Agencies (HHAs) report data from the Outcome and Assessment Information Set (OASIS) as a condition of participation from HHAs. OASIS data is used to define the quality of HHAs and need for improvement. OASIS data helps measure the rates for use of specific evidenced-based care processes in order to promote best practices across the home health industry[5].

1.1.4 Polypharmacy among home health patients

Home health patients are typically prescribed multiple medications. Further, older adults are at a higher risk of emergency hospitalizations due to adverse drug reactions[6]. This has major consequences. For example, the national average for percent of long-stay nursing home residents experiencing one or more falls with major injury is 3.2%[7]. On average, the hospitalization cost for a fall injury is $34,294 (in 2012 dollars).

1.1.5 Benefits of pharmacogenetic testing

Pharmacogenetic testing is a new, evolving area of research and has the potential to predict and reduce unnecessary medication related adverse events. Drug and gene interactions cause approximately 34% of significant interaction warnings that can lead to side effects[8]. This study will provide insight into the clinical utility of these tests. The value of pharmacogenetic testing to optimize patient outcomes has been demonstrated in psychiatry[9].

Ideally, in addition to considering a polypharmacy patient's existing burden of medications, a clinician would be able to also consider the patient's genetics. To factor in all of the information, however, is challenging. Sophisticated software is increasingly able to calculate the multitude of data points pertaining to medications and genetics. In order to do so, clinicians would need to obtain genetic testing and utilize software to generate

[3] L Hampton, et al, Emergency Department Visits by Adults for Psychiatric Medication Adverse Events, Journal of the American Medical Association: Psychiatry, July 2014.
[4] Acumen, LLC, Medication Therapy Management in Chronically Ill Populations: Final Report, http://innovation.cms.gov/Files/reports/MTM_Final_Report.pdf
[5] http://www.cms.gov/Medicare/Quality-Initiatives-Patient-Assessment-Instruments/OASIS/index.html?redirect=/oasis/01_overview.asp
[6] D Budnitz, et al, Emergency Hospitalizations for Adverse Drug Events in Older Americans, The New England Journal of Medicine, v365, n21, November 2011.
[7] Roudsari BS, Ebel BE, Corso PS, Molinari, NM, Koepsell TD. The acute medical care costs of fall-related injuries among the U.S. older adults. Injury, Int J Care Injured 2005;36:1316-22.
[8] Verbeurgt P, Mamiya T, Oesterheld J. How common are drug and gene interactions? Prevalence in a sample of 1143 patients with CYP2C9, CYP2C19 and CYP2D6 genotyping. Pharmacogenomics (2014) 15(5), 655–665.
[9] J Fagerness, et al, Pharmacogenetic-Guided Psychiatric Intervention Associated With Increased Adherence and Cost Savings, American Journal of Managed Care, v20, n5, May 2014.

recommendations regarding care. Currently the testing is used sporadically, typically unaccompanied by software analysis. To effect this change, data is needed to support the utility of the testing. The proposed study must be conducted in order to obtain the necessary data.

Pharmacogenetic testing, in combination with drug and gene interaction risk analysis software such as the YouScript® Personalized Prescribing System, can help identify the following MRPs: Improper drug selection, sub-therapeutic dosage, overdose, drug interactions, and adverse drug reactions.

1.1.6 YouScript Personalized Prescribing System

Genelex Corporation is a leader in comprehensive medication management based on the YouScript® clinical decision support tool and DNA drug sensitivity testing. The purpose of the YouScript® personalized prescribing system is to improve patient outcomes by decreasing the risk of adverse drug reactions (ADRs) and therapeutic failures caused by drug-drug, drug-gene and drug-drug-gene interactions. ADRs prevented by the YouScript® system include overdose toxicity and treatment failure caused by individual variation in pharmacokinetics caused by patient drug regimens and genetics. The YouScript® system synthesizes decades of publicly funded basic and clinical research to provide actionable information to clinicians treating patients taking multiple medications.

Controlling prescription drug treatment risk is complex because more than 85% of patients have significant genetic variation in the cytochrome p450 genes that metabolize the majority of the most commonly prescribed medications. Genetic variability in a patient's ability to metabolize many drugs can increase the risk of an ADR and impact treatment effectiveness. Drug-related problems (DRPs) are a major healthcare burden with an estimated cost of $289 billion per year in added health care costs[10]. Elderly patients, in particular, are at greater risk of an ADR[11], as they are often on multiple medications. Two-thirds of adults over age 65 use one or more prescription drugs daily[12,13,14]. Patients aged 60 years and older account for 51% of the deaths from ADRs[15,16]. Although this age group represents about 17% of the U.S. population, it accounts for 39% of hospitalizations. Ten to 17% of hospitalizations of older patients are directly related to ADRs[17]. Upon discharge, 50% of patients with ADRs experienced

---

[10] Thinking Outside the Pillbox: A System-wide Approach to Improving Patient Medication Adherence for Chronic Disease A NEHI Research Brief – August 2009 (http://www.nehi.net/writable/publication_files/file/pa_issue_brief_final.pdf)

[11] Hajjar ER, Hanlon JT, Artz MB et al. Adverse Drug Reaction Risk Factors in Older Outpatients. American Journal of Geriatric Pharmacotherapy. December 2003;1(2):82-89.

[12] Qato DM, Alexander GC, Conti RM, Johnson M, Schumm P, Lindau ST. Use of prescription and over-the-counter medications and dietary supplements among older adults in the United States. JAMA. 2008;300(24):2867.

[13] Hajjar ER, Cafiero AC, Hanlon JT. Polypharmacy in elderly patients. Am J Geriatric Pharmacotherapy. 2007. 5:345-51.

[14] Safran DG, Neuman P, Schoen C, et al. Prescription drug coverage and seniors: findings from a 2003 national survey. Health Affairs, vol. 5, pp. 152–166, 2005.

[15] Lazarou J, Pomeranz BH, Corey PN. Incidence of Adverse Drug Reactions in Hospitalized Patients: A Meta-analysis of Prospective Studies. JAMA. 1998;279(15):1200-1205.

[16] Sikdar KC, Dowden J, Alaghehbandan R, MacDonald D, Peter P, Gadag V. Adverse Drug Reactions in Elderly Hospitalized Patients: A 12-Year Population-Based Retrospective Cohort Study.

[17] Budnitz DS, Lovegrove MC, Shehab N, Richards CL. Emergency Hospitalizations for Adverse Drug Events in Older Americans. New England Journal of Medicine. November 24, 2011;365(21):2002-2012.

a decline in one or more activities of daily living, compared to 24% of patients without ADRs[18]. YouScript® guided pharmacogenetic testing assesses genetic variants in enzymes that metabolize more than 75% of routinely prescribed medications. Available tests include five enzymes in the Cytochrome P450 (CYP) group (CYP2D6, 2C9, 2C19, 3A4, AND 3A5), VKORC1, 5HTT, Factor 2, Factor 5, and others.

YouScript® offers unique advantages over other commercially available pharmacogenetic testing products. For example, it combines drug-drug interaction analysis along with drug-gene and drug-drug-gene interaction risk analysis. Further, the presentation of results and comprehensiveness of interpretation is superior to other products.

1.2 Rationale

It is not known whether referrals for YouScript® testing, which can identify MRPs, in fact prevents ADRs or otherwise reduces risk of harm among home health patients. The researchers seek to determine whether they could detect improvements in medication-related Medicare quality measures before and after initiating the YouScript® Personalized Prescribing System in home health care patients at high risk of genetically based adverse drug events.

1.3 Hypotheses

The investigator hypothesizes that patients who undergo testing via the YouScript® Personalized Prescribing System, relative to those who do not get testing, will return the hospital and emergency department less often.

2 STUDY DESIGN & OBJECTIVES

The open-label study is designed as a pilot prospective, randomized controlled trial to assess the impact of the YouScript® Personalized Prescribing System on decreased re-hospitalizations, ED visits, falls, and improvement in select OASIS metrics and clinical decision-making. The decision to utilize the YouScript® Personalized Prescribing System and all treatment decisions will be made in accordance with usual care practice, and will be made prior to the decision to participate in the study.

Outcomes will be compared between a group of prospectively enrolled patients undergoing CYP2D6, CYP2C19, CYP2C9, VKORC1, CYP3A4, and CYP3A5 testing with the YouScript® Personalized Prescribing System at the discretion of their clinician (i.e. "tested" patients) and a group of similar patients at home health meeting the same enrollment criteria (excluding the YouScript® testing) and matched on key characteristics to the tested patients using propensity score matching.

The study aims to initially enroll 100 patients from a single home health agency in the United States. The study includes 2 study arms.

Arm	Sample size	Treatment	Treatment

[18] Gray SL, Sager M, Lestico MR, Jalaluddin M. Adverse Drug Events in Hospitalized Elderly. Journal of Gerontology, 1998, vol 53A, No. 1, M59-M63.

Intervention	50	Tested	YouScript®
Controls	50	Not tested	Treatment as usual (i.e. standard drug information resources)

2.1 Primary objective

To assess the impact of pharmacogenetic testing and YouScript® Personalized Prescribing System on these outcomes at 30 and 60 days post discharge:

- The number of re-hospitalizations
- The number of Emergency Department visits

2.2 Exploratory:

To assess the impact of pharmacogenetic testing and YouScript® Personalized Prescribing System on these outcomes at 30 and 60 days post discharge:

- Time to first re-hospitalization
- Time to first ED visit
- Overall status according to OASIS M1034
- Frequency of pain according to OASIS M1242
- Frequency of confusion according to OASIS M1710
- Frequency of anxiety according to OASIS M1720
- Depression according to PHQ-2
- Frequency of disruptive behavior according to OASIS M1745
- Frequency of ADL and IADL assistance according to OASIS M2110
- The number of falls (at the end of study)
- Acceptance by the study pharmacist and passing on to clinicians (at the end of study)
- The proportion of study pharmacist recommendations acted on by clinicians (at the end of study)

3 STUDY PROCEDURES

3.1 Inclusion Criteria

- Age 65 or older.
- Willing and able to provide informed consent for study participation either directly or by a legally authorized representative (LAR).
- Presently taking or beginning treatment with at least one of the following

oral forms of medication (excluding medications taken PRN) (generic name given with major U.S. brand name given in parentheses). These medications are subject to significant drug-gene interactions as defined by FDA boxed warning, FDA cautionary labeling, clinical literature or a YouScript® algorithm-predicted significant effect:

Amitriptyline (Elavil)	Fluoxetine (Prozac)	Pimozide (Orap)
Aripiprazole (Abilify)	Flurbiprofen (Ansaid)	Piroxicam (Feldene)
Atomoxetine (Strattera)	Fluvoxamine (Luvox)	Proguanil [(Malarone (combo)]
Carvedilol (Coreg)	Haloperidol (Haldol)	Propafenone (Rythmol)
Celecoxib (Celebrex)	Hydrocodone	Propranolol (Inderal)
Citalopram (Celexa)	Ibuprofen (Motrin)	Risperidone (Risperdal)
Clobazam (Onfi)	Iloperidone (Fanapt)	Sertraline (Zoloft)
Clomipramine (Anafranil)	Imipramine (Tofranil)	Tetrabenazine (Xenazine)
Clopidogrel (Plavix)	Indomethacin (Indocin)	Thioridazine (Mellaril)
Clozapine (Clozaril)	Meloxicam (Mobic)	Timolol (Apotimol)
Codeine [Tylenol #3 (combo)]	Metoprolol (Toprol XL)	Tolterodine (Detrol)
Desipramine (Norpramin)	Mexiletine (Mexitil)	Torsemide (Demadex)
Dextromethorphan (Delsym)	Nortriptyline (Pamelor)	Tramadol (Ultram)
Diazepam (Valium)	Omeprazole (Prilosec)	Trimipramine (Surmontil)
Doxepin (Sinequan)	Oxycodone (Oxycontin)	Venlafaxine (Effexor)
Escitalopram (Lexapro)	Paroxetine (Paxil)	Voriconazole (Vfend)
Esomeprazole (Nexium)	Perphenazine (Trilafon)	Vortioxetine (Brintellix)
Fesoterodine (Toviaz)	Phenobarbital (Luminal)	Warfarin (Coumadin)
Flecainide (Tambocor)	Phenytoin (Dilantin)	

3.2 Exclusion Criteria

Patients meeting any of the following criteria will not be eligible for enrollment/inclusion in the study (ICD9 codes are included in parentheses):

- Previous CYP testing (CPT codes 81225, 81226, 81227, 81355, 81401)
- History of organ transplant (199.2; 238.77; 414.06; 414.07; 996.80-996.89; E878.0; V42.0-V42.7; V42.81-V42.84; V42.89; V42.9; V45.87; V49.83; V58.44)
- Current malabsorption syndrome (579.0), including the following:
    - Intestinal malabsorption (579.8, 579.9)
    - Postoperative malabsorption (579.3)
    - Short bowel syndrome (579.3)
- Treatment of invasive solid tumors or hematologic malignancies in the last year, excluding in situ cancers or non-melanoma skin cancer (basal cell carcinoma)
- End Stage Renal Disease (ESRD)
- Persistent acute renal failure: complete loss of kidney function >4 weeks (requiring dialysis)

- Renal failure by:
  - Glomerular filtration rater (GFR): SCr > 3 times baseline or GFR decreased 75% or SCr ≥4 mg/dL; acute rise ≥0.5 mg/dL

    OR
  - Urine Output (UO): UO < 0.3 mL/kg/h 24 h (oliguria) or anuria 12 h

3.3 Recruitment plan

A pharmacist and/or pharmacy students perform(s) chart reviews for every new patient entering home health and recruitment will come from this allotment of patients. PI will begin screening potential candidates based on these initial chart reviews and using the YouScript® risk analysis tool described in *Section 3.4.1.*

3.3.1 Screening & eligibility assessment

- Review medical and medication history to determine eligibility based on inclusion/exclusion criteria.
- Obtain and document consent from potential subject on consent form.
- Collect buccal swab for genetic testing (tested group only).

3.3.2 Enrollment/Baseline

Any new data will be collected during regularly scheduled nursing visits. Principal investigator will review to verify that subjects continue to meet the eligibility criteria.

- Obtain and document consent from subject on study consent form
- Verify inclusion/exclusion criteria
- Obtain demographic information, medical history, medication history, alcohol, and tobacco use history
- Collect blood/urine/saliva/other specimen
- Record results of selected OASIS metrics and PHQ-2
- For those randomized to intervention:
  - Collect buccal swab for testing

3.4 Randomization Procedures

Prior to enrollment the treatment assignments were generated as a simple random sample, with 50 patients in each treatment arm. The randomization was carried out in R version 3.1.1 (Vienna, Austria).

Patients will be numbered chronologically by date of enrollment and each number has been randomized *a priori* to a treatment arm as stated above. Nurses are assigned patients based on coverage area and schedule. Only the PI will have access to the randomization list as to avoid any manipulation of patients to different treatment arms.

3.4.1 YouScript® Personalized Prescribing System

The risk for a drug-drug, drug-gene, or drug-drug-gene interaction will be determined at baseline for each tested patient by conducting a risk analysis using the YouScript® risk analysis tool. The risk analysis tool uses an algorithm based on the baseline list of medications being taken by the patient in combination with the U.S. gene frequencies for CYP2D6, CYP2C9, CYP2C19, CYP3A4, CYP3A5 to produce the number of known drug-drug interactions, potential additional drug-gene and drug-drug-gene interactions, and the percentage likelihood that at least one more major interaction will be revealed with testing. In addition to matching on other variables, the risk analysis tool will be used on the untested group as well.

At the time of this writing, the standard panel of tests included in the YouScript® Personalized Prescribing System includes PCR based assays to detect the following alleles, including all common and most rare variants with known clinical significance at analytical sensitivity and specificity >99%:

- CYP2C19: active *1; inactive *2, *3, *4, *5, *6, *7, *8, *12; partially active *9, *10; rapid *17
- CYP2D6: active *1, *2; *2A, *35; inactive *3, *4, *5, *6, *7, *8, *10, *11, *12, *14, *15, *19, *20, *36; partially active *9, *17, *29, *41; gene duplications *1, *2, *4, *10, *41
- CYP2C9: active *1; inactive *2, *3, *4, *5, *6, *8, *11, *13, *15
- VKORC1: high sensitivity 1639G>A
- CYP3A4: active *1; partially active *22
- CYP3A5: active *1; inactive *3

The report provided to the clinician includes the tests conducted, the patient's phenotype for each gene tested (poor metabolizer, intermediate metabolizer, normal metabolizer, rapid metabolizer, ultra rapid metabolizer), the genotype for each gene tested, type and time of sample collection, the medications the patient is taking, the prescribing suggestions (including change, consider, monitor), the type of interaction (e.g., drug/gene, drug/drug/gene, drug/drug), interpretation of the results, and the clinical indication for testing.

The genetic test will be performed by Genelex whereas all other procedures are performed in accordance with usual care practice.

3.4.2 Drug Information Resources (for controls)

Standard drug information resources will be used (e.g., Lexicomp Online) in accordance with usual care practice. These systems contain such knowledge as drug-drug and drug-disease interactions, minimum and maximum dosing suggestions, drug-allergy cross-sensitivity groupings, and groupings of medications by therapeutic class.[19]

[19] Kuperman GJ, Reichley RM, Bailey TC. Using commercial knowledge bases for clinical decision support: opportunities, hurdles, and recommendations. J Am Med Inform Assoc. 2006;13(4):369–371.

3.5 Retention Plan

Subjects who are enrolled in the study and are discharged from home health prior to the initial 60 days will be contacted via telephone to encourage retention.

3.6 Subject Withdrawal

Subjects are free to withdraw from participation in the study at any time upon request.

An investigator may terminate a study subject's participation in the study if:

- Any clinical adverse event (AE), laboratory abnormality, or other medical condition or situation occurs such that continued participation in the study would not be in the best interest of the subject.
- The subject meets an exclusion criterion (either newly developed or not previously recognized) that precludes further study participation.

If subjects are still under care of home health then the investigator will continue to make drug therapy recommendations per usual standard of care.

New patients will be enrolled to replace withdrawn subjects, continuing chronological enrollment numbering, and will be randomized according to the randomization code.

This study may be suspended or prematurely terminated if there is sufficient reasonable cause. Written notification, documenting the reason for study suspension or termination, will be provided by the suspending or terminating party to investigator and regulatory authorities.

If the study is prematurely terminated or suspended, the principal investigator will promptly inform the IRB and will provide the reason(s) for the termination or suspension.

Circumstances that may warrant termination include, but are not limited to:

- Determination of unexpected, significant, or unacceptable risk to subjects.
- Insufficient adherence to protocol requirements.
- Data that is not sufficiently complete and/or evaluable.
- Determination of futility.

4 ETHICAL CONSIDERATIONS

4.1 Statement of compliance

The study will be conducted in accordance with the International Conference on Harmonisation guidelines for Good Clinical Practice (ICH E6), the Code of Federal Regulations on the Protection of Human Subjects (45 CFR Part 46). All personnel involved in the conduct of this study have completed human subjects protection training.

4.2 Risks

Genetic testing poses no risk to the patient.

4.3 Benefits

Potential benefits include therapeutic treatment levels, decreased adverse effects, decreased hospitalizations and emergency department visits, decreased pill burden, decreased cost to patients and healthcare systems, and improvement in quality of life.

4.4 Informed Consent Process

Extensive discussion of risks and possible benefits of study participation will be provided to subjects and their families, if applicable. A consent form describing in detail the study procedures and risks will be given to the subject. Consent forms will be IRB-approved, and the subject is required to read and review the document or have the document read to him or her. The investigator or designee will explain the research study to the subject and answer any questions that may arise. The subject will sign the informed consent document prior to any study-related assessments or procedures. Subjects will be given the opportunity to discuss the study with their surrogates or think about it prior to agreeing to participate. They may withdraw consent at any time throughout the course of the study. A copy of the signed informed consent document will be given to subjects for their records. The rights and welfare of the subjects will be protected by emphasizing to them that the quality of their clinical care will not be adversely affected if they decline to participate in this study.

The consent process will be documented in the clinical or research record.

4.5 Subject Confidentiality

Subject confidentiality is strictly held in trust by the investigator, study staff, and the sponsor and their agents. This confidentiality is extended to cover testing of biological samples and genetic tests in addition to any study information relating to subjects.

The study protocol, documentation, data, and all other information generated will be held in strict confidence. No information concerning the study or the data will be released to any unauthorized third party without prior written approval of the sponsor.

The study monitor or other authorized representatives of the sponsor may inspect all study documents and records required to be maintained by the investigator, including but not limited to, medical records (office, clinic, or hospital) for the study subjects. The clinical study site will permit access to such records.

4.6 Institutional Review Board

The protocol, informed consent form(s), recruitment materials, and all subject materials will be submitted to the Harding University IRB for review and approval. Approval of both the protocol and the consent form must be obtained before any subject is enrolled. Any amendment to the protocol will require review and approval by the IRB before the changes are implemented in the study.

4.7 Reporting Unanticipated Problems to the IRB

The Office for Human Research Protections (OHRP) considers unanticipated problems involving risks to subjects or others to include, in general, any incident, experience, or outcome that meets **all** of the following criteria:

- unexpected in terms of nature, severity, or frequency given (a) the research procedures that are described in the protocol-related documents, such as the IRB-approved research protocol and informed consent document; and (b) the characteristics of the subject population being studied;
- related or possibly related to participation in the research ("possibly related" means there is a reasonable possibility that the incident, experience, or outcome may have been caused by the procedures involved in the research); and
- suggests that the research places subjects or others at a greater risk of harm (including physical, psychological, economic, or social harm) than was previously known or recognized.

4.8 Unanticipated Problem Reporting to IRB

Incidents or events that meet the OHRP criteria for unanticipated problems require the creation and completion of an unanticipated problem report form. OHRP recommends that investigators include the following information when reporting an adverse event, or any other incident, experience, or outcome as an unanticipated problem to the IRB:

- Appropriate identifying information for the research protocol, such as the title, investigator's name, and the IRB project number;
- A detailed description of the adverse event, incident, experience, or outcome;
- An explanation of the basis for determining that the adverse event, incident, experience, or outcome represents an unanticipated problem;
- A description of any changes to the protocol or other corrective actions that have been taken or are proposed in response to the unanticipated problem.

To satisfy the requirement for prompt reporting, unanticipated problems will be reported using the following timeline:

- Unanticipated problems that are serious adverse events will be reported to the IRB within 1 week of the investigator becoming aware of the event.
- Any other unanticipated problem will be reported to the IRB within 2 weeks of the investigator becoming aware of the problem.
- All unanticipated problems should be reported to appropriate institutional officials (as required by an institution's written reporting procedures), the supporting agency head (or designee), and OHRP within one month of the IRB's receipt of the report of the problem from the investigator.

5 DATA MANAGEMENT CONSIDERATIONS

5.1 Outcome measures

The following outcome measures will be recorded by the nurse upon patient admission, Day 30, and Day 60. PI will extract the data from patient charts and manually enter the data into the data entry form (Appendix C):

Endpoint	Indicator	Scale
Overall status	OASIS M1034	0 - The patient is stable with no heightened risk(s) for serious complications and death (beyond those typical of the patient's age).<br>1 - The patient is temporarily facing high health risk(s), but is likely to return to being stable without heightened risk(s) for serious complications and death (beyond those typical of the patient's age).<br>2 - The patient is likely to remain in fragile health and have ongoing high risk(s) of serious complications and death<br>3 - The patient has serious progressive conditions that could lead to death within a year.<br>UK - The patient's situation is unknown or unclear.
Pain	OASIS M1242	0 - Patient has no pain<br>1 - Patient has pain that does not interfere with activity or movement<br>2 - Less often than daily<br>3 - Daily, but not constantly<br>4 - All of the time
Confusion	OASIS M1710	0 - Never<br>1 - In new or complex situations only<br>2 - On awakening or at night only<br>3 - During the day and evening, but not constantly<br>4 - Constantly<br>NA - Patient nonresponsive
Anxiety	OASIS M1720	0 - None of the time<br>1 - Less often than daily<br>2 - Daily, but not constantly<br>3 - All of the time<br>NA - Patient nonresponsive
Depression	PHQ-2[1]	0 – Not At all<br>1 – Several Days<br>2 – More Than Half of the Days<br>3 – Nearly Every Day
Disruptive behavior	OASIS M1745	0 - Never<br>1 - Less than once a month<br>2 - Once a month<br>3 - Several times each month<br>4 - Several times a week<br>5 - At least daily
ADL & IADL assistance	OASIS 2110	1 - At least daily<br>2 - Three or more times per week<br>3 - One to two times per week<br>4 - Received, but less often than weekly

Endpoint	Indicator	Scale
		5 - No assistance received<br>UK - Unknown [Omit "UK" option on DC]

Re-hospitalizations and dates of admission are manually recorded, by a member of the home health staff, on a white board displayed in the home health office. PI will manually enter the re-hospitalizations and time to first re-hospitalization on the data entry form for each patient.

Nurses will report ED visits, falls, and adverse drug reactions to PI as well as document in patient chart. PI will manually enter the information into the data entry form.

Additionally, for those patients who received the intervention, the study pharmacist and prescribers will be asked about impact of YouScript® testing on clinical decision-making.

5.2 Source documents and access to source data/documents

Study staff will maintain appropriate medical and research records for this study, in compliance with ICH E6, Section 4.9 and regulatory and institutional requirements for the protection of confidentiality of subjects. Study staff will permit authorized representatives of regulatory agencies to examine (and when required by applicable law, to copy) research records for the purposes of quality assurance reviews, audits, and evaluation of the study safety, progress and data validity.

5.3 Quality control and quality assurance

Details regarding procedures for ensuring high quality data will be specified in the data management plan.

6 DATA CONSIDERATIONS

Data collection and accurate documentation are the responsibility of the study staff under the supervision of the investigator. All source documents and laboratory reports must be reviewed by the study team and data entry staff members, who will ensure that they are accurate and complete. Unanticipated problems and adverse events must be reviewed by the investigator or designee.

The investigator is responsible for ensuring the accuracy, completeness, legibility, and timeliness of the data. All source documents should be completed in a neat, legible manner to ensure accurate interpretation of data. The investigator will maintain adequate case histories of study subjects, including accurate case report forms (CRFs), and source documentation.

Study documents should be retained for a minimum of 2 years. These documents should be retained for a longer period, however, if required by local regulations. No records will be destroyed without the written consent of the sponsor, if applicable. It is the responsibility of the sponsor to inform the investigator when these documents no longer need to be retained.

7 STATISTICAL CONSIDERATIONS

The statistical analysis will include the comparison of the YouScript® and control groups the primary and exploratory outcomes. Poisson regression will be used to compare the number of re-hospitalizations at 30 days, the number of re-hospitalizations at 60 days, the number of ED visits at 30 days, the number of ED visits at 60 days and the number of falls. The log-rank test will be used to compare the time to the first re-hospitalization and the time to the first ER visit. OASIS and PHQ-2 scores will be compared using the Wilcoxon rank sum test. The chi-squared test or the Fisher's exact test (as needed) will be used to compare the proportion of cases with acceptance by the study pharmacist and passing on to clinicians and the proportions of recommendations acted on by clinicians. Calculations will be carried out in R (Vienna, Austria). All tests will be two-sided. $P<0.05$ will be used to denote statistical significance. As the study is designed as a pilot, tests will not be adjusted for multiple comparisons.

8 RESULTS DISSEMINATION

The study will be registered in the public trials registry *ClinicalTrials.gov*. After the results have been analyzed a manuscript will be submitted for publication within 1 year of the last patient being enrolled in the study.

9 APPENDICES

- Appendix A: Schedule of procedures table
- Appendix B: Consent form
- Appendix C: Example Data Entry Form
- Appendix D: YouScript® Risk Analysis Tool

APPENDIX A: SCHEDULE OF PROCEDURES

Study-Specific Procedures	Screening	Baseline (Day 0)	Follow-up (Day 30)	Follow-up (Day 60)
Signed Consent Form	X			
Verification of Eligibility	X			
YouScript® testing		X		
Assess OASIS M1034/Overall status*			X*	
Assess OASIS M1242/Pain*			X*	
Assess OASIS M1710/Confusion*			X*	
Assess OASIS M1720/Anxiety*			X*	
Assess PHQ-2/Depression*			X*	
Assess OASIS M1745/Disruptive Behavior*			X*	
Assess OASIS M2110/Need for ADL & IADL Assistance*			X*	
Abstraction of data from patient chart regarding healthcare resource utilization**			X	X
Survey of clinician regarding impact on clinical decision-making			X	X

**All OASIS indicators are assessed as part of standard of care at baseline and follow-up (Day 60). Additionally, study participants will be assessed at 30 days for the indicated select OASIS indicators.*
***Falls will be assessed at Day 60; re-hospitalizations and ED visits at Day 30 and Day 60; time to 1st ED visit and re-hospitalization at time of occurrence.*

Informed Consent Form

TITLE: A Pilot Prospective, Randomized Controlled Trial Assessing the Clinical Impact of Integrated Pharmacogenetic Testing on Selected OASIS Metrics, Re-hospitalizations and Emergency Department visits

Principal Investigator: Lindsay Elliott, Pharm.D., CGP

Participant's Printed Name: ____

INTRODUCTION

We invite you take part in a pilot trial to assess the clinical impact of pharmacogenetic testing. The purpose of this study is to see if particular genetic tests might help doctors with patients who take lots of medications select the best medications based on their patients' unique DNA. Further, this research is being done because Medicare administrators and some insurance companies need more information about how particular genetic tests help doctors choose the best medications. Taking part in this study is entirely voluntary. We urge you to discuss any questions about this study with the staff members. If you decide to participate, you must sign this form to show that you want to take part.

Section 1. What is genetic testing?

DNA is the material in nearly every cell in the body, which governs the inheritance of eye color, hair color, and many other human traits. Genetic testing is done when laboratories analyze a person's unique DNA to learn things about the person. There are many kinds of genetic tests. Only a handful of specific genetic tests will be done in this study. The genetic tests in this study may give information to patients and their doctors about how one's body processes certain medications. This information may help a doctor prescribe the medication and dose that is the most effective for a patient with the least negative side effects. Only some patients enrolled in this study will undergo these tests.

Section 2. What is the purpose of the research?

In this research study, health information will be compared between patients who receive specific genetic tests and those that did not in order to find out the following:

- Whether patients who have specific genetic tests may return to the hospital or visit the emergency department less often than patients who do not.
- Whether patients who have specific genetic tests have less adverse drug reactions, falls, and other selected metrics than patients who do not.

Section 3. What procedures will be done?

Procedures limited only to participants assigned to genetic testing:
After a patient agrees to participate in the study, if he or she is assigned to receive certain genetic tests, then a clinician will run a swab inside the patient's cheek to gather a sample for DNA processing. The sample will be sent to the Genelex Corporation laboratory in Seattle, WA.

<u>All study participants:</u>
You will be assessed 30 days after entering the study on select metrics that are identical to part of the full assessment done for all patients when they enter home health and at discharge. Additionally, information available from your insurance company, hospital, and clinician will be gathered related to how often you saw the doctor, whether you were hospitalized, and whether you visited the Emergency Room.

Section 4. Can I receive other treatments?

You may choose not to participate in this study. Your medication counseling session will be done per standard approaches based on existing health information.

Section 5. How long will the study last?

The researchers will gather data for two months after a patient agrees to join the study.

Section 6. What are my risks?

The risks of this study are minimal. You may feel upset thinking about or talking about personal information related to genetic testing. These risks are similar to those you experience when discussing personal information with others. If you feel upset from this experience, you can tell the researcher, and he/she will tell you about resources available to help.

The results of this test will only be shared with your clinician, nurse, and a pharmacist.

Section 7. What are the potential benefits?

We cannot promise any direct benefit for taking part in this study. However, possible benefits include knowing your genetic test results and receiving the most effective medications with the least amount of adverse effects based on your DNA. We hope the information we get from this study may help develop a greater understanding of genetic testing in the future.

Section 8. How will my confidentiality be protected?

We will do everything we can to keep your information. Study information will be kept in a secured manner and electronic records will be password protected. Results from the genetic information will be kept confidential and will not be shared with anybody except your clinician, nurse, and a pharmacist. We may also need to disclose information if required by law.

In order to conduct this study and make sure it is conducted as described in this form, the research records may be used and reviewed by others who are working with us on this research:

- Members of the research team at Harding University and Genelex;
- The Harding University Institutional Review Board (IRB), who reviews research involving people to make sure the study protects your rights

If we share your information with groups outside of Harding University or Genelex, we will not share your name or identifying information. We will label your information with a code number, so they will

not know your identity.
If you do not want us to use information about your health, you should not be part of this research. If you choose not to participate, you can still receive Medication Therapy Management Services through your home health agency.

Section 9. What is the cost/compensation for participation?

There are no costs and you will not be compensated for participation. Your medical insurance will be billed for any care you receive during the research study, including the cost of the test.

Section 10. How is this pilot study funded?

Harding University

The Genelex Corporation will compensate the investigator for her effort working on this study. Genelex operates and maintains the clinical decision support software referred to as the YouScript Personalized Prescribing System. The Genelex Corporation also performs the genetic tests. The genetic tests are not experimental. The Genelex Corporation maintains a laboratory, which is accredited, certified, and licensed to perform testing in all U.S. states. Genelex hopes to see if patients benefit from the test by comparing health data between patients who have the test and those that do not.

Section 11. Is participation voluntary?

Research studies include only people who choose to take part. You can tell us that you do not want to be in this study. You can start the study and then choose to stop the study later. This will not affect your relationship with the investigator. Participation is voluntary. Refusal to participate will involve no penalty or loss of benefits to which you are otherwise entitled. You may discontinue participation at any time without any penalty or loss to benefits.

Section 12. Who should I contact for information, questions, or concerns?

Contact the Institutional Review Board (IRB) if you have questions regarding your rights as a research participant. Also, contact the IRB if you have questions, complaints or concerns, which you do not feel you can discuss with the investigator. The Harding University IRB may be reached by phone at (501) 279-4640 or by e-mail at *irb@harding.edu*.

(CONTINUED NEXT PAGE)

AUTHORIZATION FOR USE OF YOUR PROTECTED HEALTH INFORMATION

Signing this document means you allow us, the researchers in this study, and others working with us to use some information about your health for this research study.

This is the information we will use and include in our research records:

- Demographic and identifying information like your name, date of birth, gender, medication list
- Related medical information about you like your current and past medical conditions, family medical history, medication allergies
- All laboratory tests that will be done in the study

What if I decide to Not Participate after I sign the Consent Form?

You can tell us anytime that you do not want to be in this study and do not want us to use your health information. You can also tell us in writing. If you change your mind, we will not be able to collect new information about you, and you will be withdrawn from the research study. However, we can continue to use information we have already started to use in our research, as needed to maintain the integrity of the research.

This authorization does not have an expiration date.

CONSENT:

Before making the decision regarding enrollment in this study you should have:

- Discussed this study with the investigator or designee,
- Reviewed the information in this form, and
- Had the opportunity to ask any question you may have.

Your signature below means that you have received this information, have asked the questions you currently have about the study and those questions have been answered. You will receive a copy of the signed and dated form to keep for your future reference.

Participant: I agree to participate in this research study and authorize you to use and disclose health information about me for this study, as you have explained in this document.

____ ____________

Participant's Signature Date

Person Explaining the Pilot Clinical Study: Your signature below means that you have explained the study to the participant or parent/guardian of minor participant and have answered any questions he/she has about the study.

____

Name of Person Obtaining Authorization and Consent

____ ____________

Signature of Person Obtaining Authorization and Consent Date

APPENDIX C: Example Data Entry Form

				M1034/Overall Status			M1242/Pain			M1710/Confusion			M1720/Anxiety			M1745/Behavior			M2110/ADL & IADL			PHQ2/Depression			Hospitalizations			ER Visits		
PATIENT	AGE	ETHNICITY	GENDER	Admit	Day 30	Day 60	Admit	Day 30	Day 60	Admit	Day 30	Day 60	Admit	Day 30	Day 60	Admit	Day 30	Day 60	Admit	Day 30	Day 60	Admit	Day 30	Day 60	Time to 1st	# at Day 30	# at Day 60	Time to 1st	# at Day 30	#
1																														
2																														
3																														
4																														
5																														
6																														
7																														
8																														
9																														
10																														
11																														
12																														
13																														
14																														
15																														
16																														
17																														
18																														
19																														
20																														

APPENDIX C: YouScript® Risk Analysis Tool

youScript Personalized Prescribing System

Adverse Drug Reaction Risk Assessment

Which medical conditions apply to you?

- ☐ Mental Health Condition
- ☐ Asthma / CPOD
- ☐ Diabetes
- ☐ Cholesterol (High)
- ☐ Organ Transplant
- ☐ Acid Reflex
- ☐ Thyroid Disorder
- ☐ Blood Pressure (High)
- ☐ Migraines
- ☐ Enlarged Prostate
- ☐ Arthritis
- ☐ Depression
- ☐ Osteoporosis
- ☐ Peptic Ulcer
- ☐ HIV

3 points per condition *(Automatically high risk if 5 or more conditions)* — Total:

How many prescription medications do you take regularly?
☐ None *(0 points)* ☐ 1 – 4 *(2 points)* ☐ 5+ *(Automatically high risk if taking 4 or more)* — Total:

Does more than one physician prescribe medicine to you?
☐ Yes *(3 points)* ☐ No *(0 points)* — Total

Are you at least 65 years old?
☐ Yes *(3 points)* ☐ No *(0 points)* — Total

Have you ever experienced a bad reaction to a medication that caused you to visit the emergency room or be hospitalized?
☐ Yes *(5 points)* ☐ No *(0 points)* — Total

Have you been hospitalized in the last 30 days?
☐ Yes *(3 points)* ☐ No *(0 points)* — Total

Do you sometimes experience unwanted side effects from any of your medications?
☐ Yes *(3 points)* ☐ No *(0 points)* — Total

Do you sometimes feel that your medications are not working?
☐ Yes *(3 points)* ☐ No *(0 points)* — Total

Which of the following medications are you currently taking?

- ☐ Abilify (aripiprazole)
- ☐ Aciphex (rabeprazole)
- ☐ Anafranil (clomipramine)
- ☐ Ansaid (flurbiprofen)
- ☐ Celebrex (celecoxib)
- ☐ Celexa (citalopram)
- ☐ Clozaril (clozapine)
- ☐ codeine
- ☐ Coreg (carvedilol)
- ☐ Coumadin (warfarin)
- ☐ Detrol (tolterodine)
- ☐ Dexilant (dexlansoprazole)
- ☐ Elavil (amitriptyline)
- ☐ Evoxac (cevimeline)
- ☐ Fanapt (iloperidone)
- ☐ Lopressor (metoprolol)
- ☐ Luvox (fluvoxamine)
- ☐ Mellaril (thioridazine)
- ☐ Norpramin (desipramine)
- ☐ Nuedexta (dextromethorphan)
- ☐ Nexium (esomeprazole)
- ☐ Onfi (clobazam)
- ☐ Pamelor (nortriptyline)
- ☐ Plavix (clopidogrel)**
- ☐ Protonix (pantroprazole)
- ☐ Provigil (modafinil)
- ☐ Razadyne (galantamine)
- ☐ Risperdal (risperidone)
- ☐ Rythmol (propafenone)
- ☐ Sinequan (doxepin)
- ☐ Soma (carisoprodol)
- ☐ Strattera (atomexetine)
- ☐ Tofranil (imipramine)
- ☐ Trilafon (perphenazine)
- ☐ Tylenol #3
- ☐ Ultram (tramadol)
- ☐ Valium (diazepam)
- ☐ Vivactil (protriptyline)
- ☐ Xenazine (tetrabenazine)

5 points per medication ***** (Automatically high risk if taking Plavix or clopidogrel)*** — Total

Automatic High Risk	Your Results		Grand Total
☐ **Plavix (clopidogrel)**	☐ Low Risk	0 – 9 points	
☐ **5 or more medications**	☐ Moderate Risk	10 – 19 points	
☐ **5 or more medical conditions**	☐ High Risk	20+ points	

Patient Name______________________________**Signed**________________________

For immediate consultation Call 1-877-431-4362
Hours 7:00 AM to 6:00 PM PST, 10:00 AM to 9:00 PM EST, Fax: 206-219-4000
3101 Western Ave., Ste. 100 Seattle, WA 98121
E-mail:info@YouScript.com Web: www.YouScript.com

Version Aulinskas 4/10/13

©2013 Genelex Corporation

STATISTICAL ANALYSIS PLAN

Harding University - Home Health Trial of YouScript

Version Date:	December 16 , 2014
Study:	A Pilot Prospective, Randomized Controlled Trial Assessing the Clinical Impact of Integrated Pharmacogenetic Testing on Selected OASIS Metrics, Re-hospitalizations and Emergency Department visits
Principal Investigator:	Lindsay Elliott, PharmD, CGP
Prepared By	Moni Neradilek, The Mountain-Whisper-Light Statistics
Harding University IRB file:	IRB 2015-003

Statistical analysis plan section from the trial protocol

Taken from section number 7, "Statistical considerations"

The statistical analysis will include the comparison of the YouScript and control groups the primary and exploratory outcomes. Poisson regression will be used to compare the number of re-hospitalizations at 30 days, the number of re-hospitalizations at 60 days, the number of ED visits at 30 days, the number of ED visits at 60 days and the number of falls. The log-rank test will be used to compare the time to the first re-hospitalization and the time to the first ER visit. OASIS and PHQ-2 scores will be compared using the Wilcoxon rank sum test. The chi-squared test or the Fisher's exact test (as needed) will be used to compare the proportion of cases with acceptance by the study pharmacist and passing on to clinicians and the proportions of recommendations acted on by clinicians. Calculations will be carried out in R (Vienna, Austria). All tests will be two-sided. P<0.05 will be used to denote statistical significance. As the study is designed as a pilot, tests will not be adjusted for multiple comparisons.

March 24, 2016 Amendments to statistical plan made after study completion

1. Three outcomes were added post-hoc:

    A. Composite of re-hospitalizations, ER visits and deaths (analyzed as counts at 30 days, 60 days and time to the first event)

    B. Composite of re-hospitalizations and ER visits (analyzed as counts at 30 days, 60 days and time to the first event)

    C. Death by day 60 of the study (analyzed as binary)

2. Two planned outcomes were not compared:

    A. The proportion of cases with acceptance by the study pharmacist and passing on to clinicians

    B. The proportions of recommendations acted on by clinicians.

The comparison did not make sense as these two outcomes applied only to the intervention and not to the control group. In the final analysis the estimated proportions for the intervention were presented.

3. To avoid inappropriate use of Poisson regression we tested for over-dispersion in all Poisson models. In cases when the null hypothesis of no over-dispersion was rejected ($p<0.05$) we used the negative binomial regression instead of the Poisson regression. This tended to result in appropriately less over-optimistic estimates of the variance.
