# Supplementary material for: Clinical impact of pharmacogenetic profiling with a clinical decision support tool in polypharmacy home health patients: A prospective pilot randomized controlled trial
Source: PLoS One. 2017 Feb 2;12(2):e0170905. doi: 10.1371/journal.pone.0170905 (PMC5289536; doi:10.1371/journal.pone.0170905)
Supplement: S1 Appendix — (PDF) [file pone.0170905.s005.pdf]

Description of the YouScript® Clinical Decision Support Tool

YouScript® clinical decision support (CDS) tool, is an evidence-based, algorithm-driven software service that identifies all classes of medication interactions (drug-drug, drug-gene, and cumulative drug and/or gene interactions). It calculates the cumulative effects of multiple interactions between prescription drugs, over the counter medications, herbal preparations, and pharmacogenomic data when available. The CDS predicts AUC changing pharmacokinetic interactions from known metabolic data such as the (Ki) of DME inhibiting and inducing drugs and percentage metabolism of drug substrates by affected enzymes. The pharmacokinetic interactions considered by the algorithm include alterations to absorption, distribution, metabolism, and excretion. Metabolism and excretion include phase 1 reactions by cytochrome P450s, esterases, and others, phase 2 reactions considered include glucuronidation and sulfation. Biochemical interference with transporters such as the ATP-binding cassette and solute carrier transporters are also taken into account. Pharmacogenetic effects on pharmacokinetics include those caused by CYP2D6, CYP2C9, CYP2C19 and many other DMEs.

A list of 1924 medications and other factors that affect patient drug levels is available for query. Patient reports are produced based on patient drug list by accessing a database of 11,044 advisory notes that include links to the 10,592 professionally curated pharmacokinetic, pharmacodynamic, and pharmacogenomic publications that form the YouScript® knowledge base. Reports identify patients for whom genetic testing could produce clinically actionable information, provide suggestions for the alteration of drug regimens, and provide lists of alternative medications by therapeutic class or indication

A more robust description of the algorithm is available from the relevant US patents (http://www.google.com/patents/US8311851; http://www.google.com/patents/US8099298). Drug dosage or hepatic or kidney function are not currently taken into account by the algorithm.

Example of the personalized prescribing report generated by the YouScript® Clinical Decision Support Tool system

For each tested patient, a personalized prescribing report was curated by a pharmacist for the patient's physician to review. A YouScript® personalized prescribing report contains the patient's genetic results, the patient's medication list, and a list of prescribing suggestions for the physician to review. An example of the personalized prescribing report generated by the YouScript® system and pharmacogenetic laboratory test report is included as an appendix below.

youscript

Personalized Prescribing Report

**Patient:**	John Doe	**Date of Birth:**	06/19/1975
**Account:**	Doe Primary Care	**Lab #:**	81338
**Referrer:**	Johnathan Doe, MD	**Reported:**	09/29/2016

CUMULATIVE DRUG-DRUG AND DRUG-GENE INTERACTIONS

Impact	Medication	Cause(s)	Effects & Management
MAJOR	codeine<br>*component of Tylenol 3*	CYP2D6 Intermediate Metabolizer<br>Paxil	• Codeine active metabolite levels may decrease by 81-100%.<br>• Decreased effectiveness of codeine.<br>• Increase codeine dose for pain control as necessary.<br>• Potential alternatives to codeine for pain include: hydromorphone (Dilaudid), morphine (MS Contin) and oxymorphone (Opana).

DRUG-DRUG INTERACTIONS

Impact	Medication	Cause(s)	Effects & Management
CONTRA-INDICATED	Zocor	gemfibrozil	• Coadministration of gemfibrozil and Zocor is contraindicated.<br>• Zocor active metabolite levels may increase by 76-200%.<br>• Increased risk of rhabdomyolysis and myopathy.<br>• Avoid coadministration of gemfibrozil and Zocor if possible.<br>• Potential alternatives to gemfibrozil include: fenofibric acid (Trilipix) and fenofibrate (Tricor).

DRUG-GENE INTERACTIONS

Impact	Medication	Cause(s)	Effects & Management
MINOR	Paxil	CYP2D6 Intermediate Metabolizer	• Paxil levels may increase by 26-75%.<br>• Increased risk of weakness, sexual dysfunction, somnolence, sweating and nausea.<br>• Decrease Paxil dose if necessary.<br>• Potential alternatives to Paxil include: vilazodone (Viibryd), mirtazapine (Remeron) and desvenlafaxine (Pristiq).

ALTERNATE MEDICATIONS BEING CONSIDERED

Impact	Medication	Cause(s)	Effects & Management
MODERATE	Celexa	CYP2C19 Poor Metabolizer	• Celexa levels may increase by 76-200%.<br>• Increased risk of dry mouth, sexual dysfunction, somnolence, QTc prolongation and nausea.<br>• Initiate Celexa dose at 50% of normal in CYP2C19 Poor Metabolizer patients.<br>• Limit Celexa dose to 20 mg daily in CYP2C19 Poor Metabolizer patients.<br>• Potential alternatives to Celexa include: vilazodone (Viibryd), mirtazapine (Remeron) and desvenlafaxine (Pristiq).

Genelex Labs LLC © 2016

ADVISORY NOTE TO TREATING PRACTITIONER:

The YouScript software and Personalized Prescribing Report are clinical decision support tools intended to add to the information healthcare practitioners have available when evaluating and prescribing medications. The recommendations provided may be based on limited patient information and do not supersede sound clinical judgement. Minor interactions and small changes in drug levels that may impact the patient are generally not reported. The healthcare practitioner has responsibility for all treatment decisions independent of the available genetic test results and any information provided by YouScript software, reports or consultations.

Reviewed By: Sample Pharmacist, PharmD
(877) 796-4362 | Genelex Labs LLC | 3101 Western Ave. Ste. 100, Seattle, WA 98121

MEDICATION HISTORY:

Current Medications:

aspirin low dose, gemfibrozil, hydrochlorothiazide, Paxil, Prinivil, Tylenol 3, Zocor

Alternate Medications:

Celexa

Failed Medications:

Zoloft

Interaction Impact Legend:

Contraindicated	This drug has an interaction that is contraindicated in the product insert due to the potential for a severe or life threatening reaction. This combination should not be administered together.
Major	This drug has an interaction that may result in severe clinical effects or large changes in drug levels. The risks of the interaction generally outweigh the benefits of prescribing the drug.
Moderate	This drug has an interaction that may result in substantial clinical effects or moderate changes in drug levels. Changes in therapy, such as making dose adjustments or prescribing alternatives, may be warranted.
Minor	This drug has an interaction that may result in minor clinical effects or small changes in drug levels. The benefits of prescribing the drug generally outweigh the risks of the interaction. Major changes in therapy are not expected, although minor dose adjustments may be appropriate.
Minimal	This drug may be associated with clinically insignificant and/or favorable interactions. No change in therapy is necessary.

genelex

Pharmacogenetic Laboratory Test Report

**Patient:**	John Doe	**Date of Birth:**	06/19/1975	**Collected:**	09/23/2016
**Account:**	Doe Primary Care	**Lab #:**	81338	**Received:**	09/26/2016
**Referrer:**	Johnathan Doe, MD	**Sample:**	Buccal Swab	**Reported:**	09/29/2016

RESULTS

Test:	Phenotype:	Genotype:
CYP2D6	Intermediate Metabolizer	*1/*4
CYP2C19	Poor Metabolizer	*2/*2
CYP2C9	Normal Metabolizer	*1/*1
CYP3A4	Normal Metabolizer	*1/*1
CYP3A5	Non-Expresser	*3/*3

LABORATORY RESULTS INTERPRETATION

CYP2D6 Intermediate Metabolizers have decreased CYP2D6 activity. For CYP2D6 inactivated drugs, consider prescribing decreased doses to prevent adverse effects. For prodrugs that require activation by CYP2D6, consider prescribing increased doses or alternative treatment for optimal therapeutic response.

CYP2C19 Poor Metabolizers have greatly decreased CYP2C19 activity. For CYP2C19 inactivated drugs, consider prescribing decreased doses or alternative treatment to prevent adverse effects. For prodrugs that require activation by CYP2C19, consider prescribing increased doses or alternative treatment for optimal therapeutic response.

CYP2C9 Normal Metabolizers have normal (extensive) CYP2C9 activity. Prescribe CYP2C9 metabolized drugs at standard doses.

CYP3A4 Normal Metabolizers have normal (extensive) CYP3A4 activity. Prescribe CYP3A4 metabolized drugs at standard doses. Patients may still have significant variation in CYP3A4 activity due to various patient and environmental factors, despite having a CYP3A4 Normal Metabolizer phenotype.

CYP3A5 Non-Expressers (also known as Poor Metabolizers) have greatly decreased CYP3A5 activity. The majority of the population (60-90%) have this genotype, except for people of African origin. Prescribe CYP3A5 metabolized drugs at standard doses.

METHODS

Clinical Indication for Testing: Patient taking medicines metabolized by the cytochrome P450s or other enzymes, has a personal or family history of adverse reactions including treatment failure, or to confirm the presence or absence of relevant genotypes and as an aid to dosing and co-medication administration. DNA testing does not replace the need for clinical and therapeutic drug monitoring.

Methodologies: PCR based assays detect listed alleles, including common and most rare variants (frequency >1%) with known clinical significance at analytical sensitivity and specificity >99%. Rare variants may not have been observed at Genelex. The absence of a positive test result for all variants listed may result in the assignment of a *1 (wild-type) status. Other known clinically significant variants not listed or not discovered are not detected and thus may lead to the possibility of loss of function or increased function in an individual with that variant being erroneously called *1. Variants tested may include: **CYP2D6**: *2,*2A,*3-*12,*14,*15,*17,*19,*20,*29,*35,*36,*41, gene deletion and duplications. **CYP2C19**: *2-*10,*12,*17. **CYP2C9**: *2-*6,*8,*11,*13,*15. **CYP3A4**: *22. **CYP3A5**: *3. Rare false negative or false positive results may occur. Genetic test results may be unclear or difficult to interpret due to current understanding of genetic disorder or condition and /or technical limitations of the test. Predicted phenotype and allele functionality may change depending upon the emergence of new literature, industry standards and guidelines. Assays developed and performance characteristics were determined by Genelex. These tests have not been cleared or approved by the US Food and Drug Administration. FDA does not require these tests to go through premarket FDA review. These tests are used for clinical purposes and should not be regarded as investigational or for research. The laboratory is regulated under CLIA as qualified to perform high-complexity clinical testing. Genelex is accredited by the College of American Pathologists (CAP 4344001), certified under the Clinical Laboratory Improvement Amendments (CLIA No. 50D0980559), Washington State Medical Test Site No. MTSA.FS.60671761, New York State Department of Health license no. PFI 8201 and is licensed to perform high complexity clinical testing in all US states. **References:** Available by request. **Liability Disclaimer:** The information provided in this report does not constitute medical advice. While this report is believed to be accurate and complete at issue, the data is provided "as is", without warranties of any kind, express or implied, including fitness for a particular purpose.

Laboratory Director: Teresa H. Aulinskas, Ph.D. | (877) 431-4362 | Genelex Labs LLC | 3101 Western Ave., Ste. 100, Seattle, WA 98121
