# Supplementary material for: Clinical impact of pharmacogenetic profiling with a clinical decision support tool in polypharmacy home health patients: A prospective pilot randomized controlled trial
Source: PLoS One. 2017 Feb 2;12(2):e0170905. doi: 10.1371/journal.pone.0170905 (PMC5289536; doi:10.1371/journal.pone.0170905)
Supplement: S1 Table — (DOCX) [file pone.0170905.s006.docx]

**S1 Table.** **Comparison of selected OASIS and PHQ-2 metrics between the two groups.**

| **Selected Metrics** | **Untested** | | | **Tested** | | | **diff.** | **p*** | |
| --- | --- | --- | --- | --- | --- | --- | --- | --- | --- |
|  | **N** | **mean (range)** | **median** | **N** | **mean (range)** | **median** |  | |  |
| Overall OASIS^ score at baseline | 52 | 2.63 (1-4) | 3 | 56 | 2.64 (1-4) | 3 | 0.01 | | 0.70 |
| Overall OASIS^ score at 30 days | 46 | 2.00 (1-3) | 2 | 55 | 2.11 (1-4) | 2 | 0.11 | | 0.61 |
| Overall OASIS^ score at 60 days | 41 | 1.76 (1-4) | 1 | 49 | 1.71 (1-4) | 2 | -0.04 | | 0.89 |
| PHQ-2^#^ score at baseline | 52 | 1.21 (1-4) | 1 | 56 | 1.14 (1-2) | 1 | -0.07 | | 0.64 |
| PHQ-2^#^ score at 30 days | 48 | 1.08 (1-2) | 1 | 56 | 1.12 (1-3) | 1 | 0.04 | | 0.67 |
| PHQ-2^#^ score at 60 days | 41 | 1.51 (1-4) | 1 | 49 | 1.41 (1-4) | 1 | -0.10 | | 0.83 |
| PHQ-2^$^ score at baseline | 52 | 1.25 (1-4) | 1 | 56 | 1.18 (1-2) | 1 | -0.07 | | 0.99 |
| PHQ-2^$^ score at 30 days | 48 | 1.06 (1-2) | 1 | 56 | 1.16 (1-3) | 1 | 0.10 | | 0.27 |
| PHQ-2^$^ score at 60 days | 41 | 1.59 (1-4) | 1 | 49 | 1.35 (1-3) | 1 | -0.24 | | 0.86 |

The scores were evaluated and documented at time of admission to home health, at 30 days, and at 60 days for improvement in overall status, pain, confusion, anxiety, depression, disruptive behavior, and the need for assistance with activities of daily living. The differences in the overall all scores at 30 days and 60 days between the tested and untested group were relatively small and not statistically significant.

diff., difference (tested-untested); p, p-value; OASIS, Outcome and Assessment Information Set; ^Overall OASIS metrics included were overall status M1034, pain M1242, confusion M1710, anxiety M1720, disruptive behavior M1745, and ADL & IADL assistance 2110; PHQ, Patient Health Questionnaire; ^#^OASIS PHQ-2 metrics - little interest ort pleasure in doing things; ^$^OASIS PHQ-2 metrics - feeling down, depressed, or hopeless; *Wilcoxon rank sum test, N, number of patients.
