# Supplementary material for: Clinical impact of pharmacogenetic profiling with a clinical decision support tool in polypharmacy home health patients: A prospective pilot randomized controlled trial
Source: PLoS One. 2017 Feb 2;12(2):e0170905. doi: 10.1371/journal.pone.0170905 (PMC5289536; doi:10.1371/journal.pone.0170905)
Supplement: S2 Table — (DOCX) [file pone.0170905.s007.docx]

**S2 Table. Comparison of patient diseases, disorders and related health problems between the two groups.**

| **Patient Diseases, Disorders and Related Health Problems** | **Patient Counts (Tested)** | **Patient Counts (Untested)** | **p*** |
| --- | --- | --- | --- |
| Ischemic heart diseases (atherosclerosis, myocardial infarction, etc.) | 42 | 41 | 0.65 |
| Hypertensive diseases | 38 | 27 | 0.09 |
| Diseases of the musculoskeletal system and connective tissue (arthropathies, dorsopathies, osteopathies, soft tissue disorders, etc.) | 34 | 33 | 0.78 |
| Congestive heart failure | 20 | 23 | 0.37 |
| Chronic obstructive pulmonary disease | 21 | 21 | 0.76 |
| Diseases of the digestive system (constipation, diverticulosis, gastro-esophageal reflux disease, hematemesis, irritable bowel syndrome etc.) | 15 | 20 | 0.20 |
| Atrial fibrillation | 19 | 19 | 0.78 |
| Diabetes mellitus | 19 | 18 | 0.94 |
| Pulmonary and other heart diseases (cardiomegaly, cardiomyopathy, endocarditis, nonrheumatic aortic valve disorder, etc.) | 14 | 16 | 0.51 |
| Pneumonia | 11 | 10 | 0.95 |
| Diseases of the nervous system (Alzheimer’s, chronic pain, encephalopathy, epilepsy, neuropathy, etc.) | 10 | 11 | 0.67 |
| Diseases of the skin (cellulitis, non-pressure chronic ulcers, pressure ulcer, psoriasis, etc.) | 10 | 9 | 0.94 |
| Cerebrovascular diseases (aphasia, dysphasia, hemiplegia following cerebral infarction) | 10 | 0 | 0.001 |
| Hypothyroidism | 9 | 9 | 0.87 |
| Hypotension | 8 | 9 | 0.67 |
| Certain infectious diseases (bacterial, viral hepatitis, etc.) | 6 | 9 | 0.32 |
| Mental and behavioral disorders (bipolar, dementia, major depressive disorder, panic disorder, etc.) | 8 | 7 | 0.90 |
| Eye diseases (low vision, macular degeneration, unspecified visual loss, etc.) | 8 | 1 | 0.03 |
| Other diseases of the respiratory system (asthma, bronchitis, unspecified respiratory failure, etc.) | 4 | 8 | 0.17 |
| Diseases of arteries, arterioles, capillaries and veins (aortic aneurysm, peripheral vascular disease, Raynaud's syndrome, venous insufficiency, etc.) | 7 | 6 | 0.88 |
| Urinary tract infection | 7 | 5 | 0.63 |
| Renal failure | 6 | 5 | 0.85 |
| Enlarged prostate and bladder dysfunction | 5 | 2 | 0.44 |
| Morbid obesity | 4 | 1 | 0.37 |
| Anemia | 2 | 0 | 0.50 |
| Malignant neoplasms (lung) | 1 | 1 | 1.00 |

p-value; * the chi-squared test or the Fisher’s exact test (as appropriate - i.e. chi-squared test is used only if the expected counts are >=5.) Statistically significant p-values are highlighted in red.
