# Supplementary material for: Clinical impact of pharmacogenetic profiling with a clinical decision support tool in polypharmacy home health patients: A prospective pilot randomized controlled trial
Source: PLoS One. 2017 Feb 2;12(2):e0170905. doi: 10.1371/journal.pone.0170905 (PMC5289536; doi:10.1371/journal.pone.0170905)
Supplement: S3 Table — (DOCX) [file pone.0170905.s008.docx]

**S3 Table. Comparison of the frequency of drugs prescribed between the two groups.**


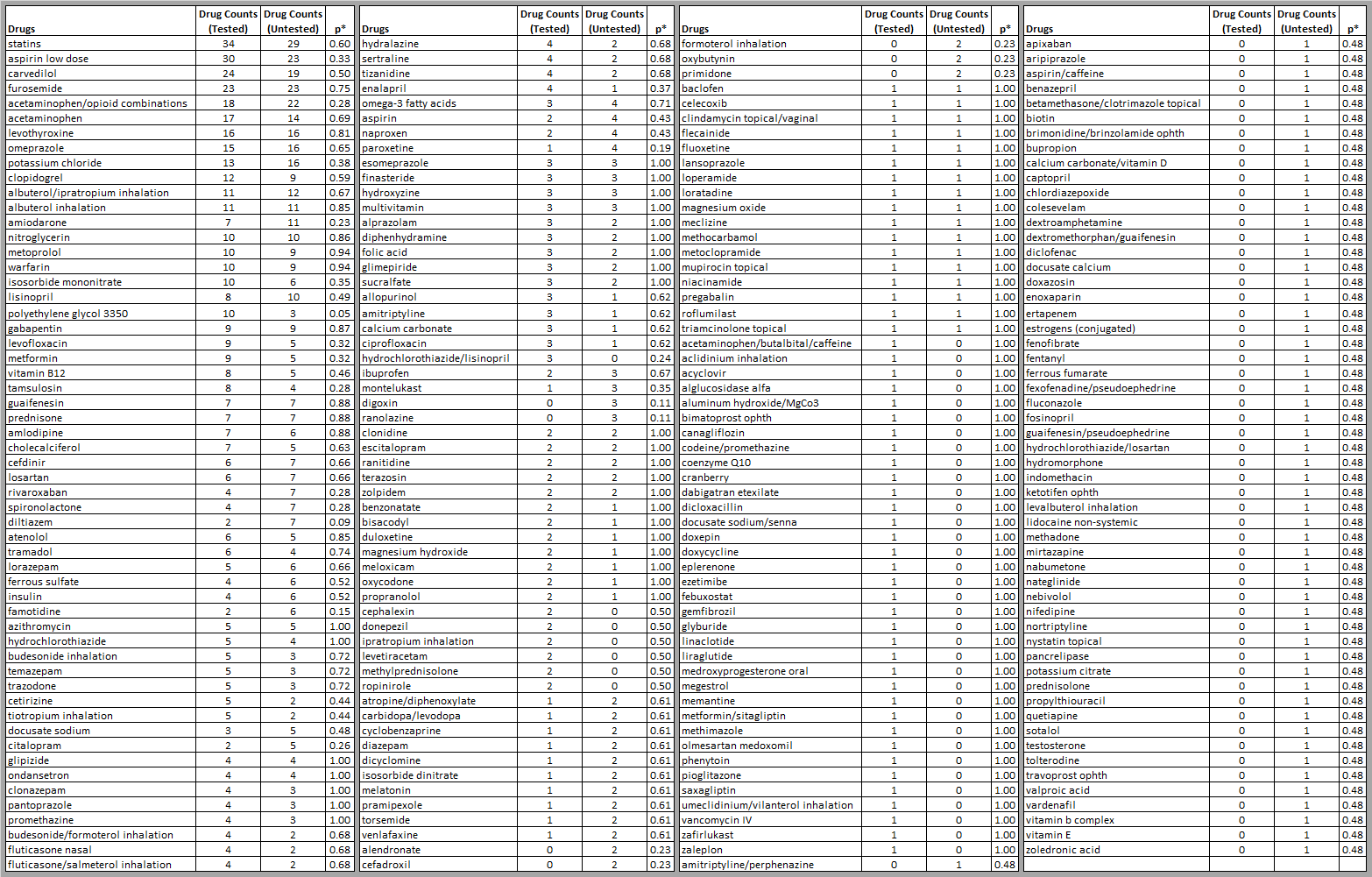
The list shows the complete list of drugs prescribed between the two groups. p-value; * the chi-squared test or the Fisher’s exact test (as appropriate - i.e. chi-squared test is used only if the expected counts are >=5).
