# Supplementary material for: Clinical impact of pharmacogenetic profiling with a clinical decision support tool in polypharmacy home health patients: A prospective pilot randomized controlled trial
Source: PLoS One. 2017 Feb 2;12(2):e0170905. doi: 10.1371/journal.pone.0170905 (PMC5289536; doi:10.1371/journal.pone.0170905)
Supplement: S4 Table — (DOCX) [file pone.0170905.s009.docx]

**S4 Table. Comparison of differences in the demographic characteristics of patients between this trial and Hocum study.**

| **Demographics** | | **Home Health Trial** | | **Hocum* et al.** | |
| --- | --- | --- | --- | --- | --- |
|  |  | **mean** | **SD** | **mean** | **SD** |
| Drug Count | | 11.6 | 4.4 | 8.2 | 6.2 |
| Age | | 75.6 | 10.7 | 60 | 18.1 |
| **Demographics** | | **Home Health Trial** | | **Hocum* et al.** | |
|  |  | **n** | **%** | **n** | **%** |
| Age | 51-64 yrs | 20 | 18.2% | 12497 | 54.4% |
|  | 65+ yrs | 90 | 81.8% | 10458 | 45.6% |
| Gender | Male | 42 | 38.2% | 9330 | 40.6% |
|  | Female | 68 | 61.8% | 12832 | 55.9% |
|  | Unknown | 0 | 0.0% | 793 | 3.5% |
| Race | African American | 1 | 0.9% | 1750 | 7.6% |
|  | Asian | 0 | 0.0% | 230 | 1.0% |
|  | Caucasian | 109 | 99.1% | 10566 | 46.0% |
|  | Hispanic | 0 | 0.0% | 2772 | 12.1% |
|  | Jewish (Ashkenazi) | 0 | 0.0% | 66 | 0.3% |
|  | Unknown | 0 | 0.0% | 7571 | 33.0% |

* Hocum BT et al. Cytochrome P-450 gene and drug interaction analysis in patients referred for pharmacogenetic testing. Am J Health Syst Pharm. 2016;73(2):61-7.

SD, standard deviation; n, number of patients; %, percentage.
