# Supplementary material for: Clinical impact of pharmacogenetic profiling with a clinical decision support tool in polypharmacy home health patients: A prospective pilot randomized controlled trial
Source: PLoS One. 2017 Feb 2;12(2):e0170905. doi: 10.1371/journal.pone.0170905 (PMC5289536; doi:10.1371/journal.pone.0170905)
Supplement: S5 Table — (DOCX) [file pone.0170905.s010.docx]

**S5 Table. Estimated financial savings from re-hospitalizations and ED visits reduction.**

| **Outcomes** | **Mean number of events per patient** | **Average cost per event** | **Average cost per patient** | **Per patient savings vs. untested** |
| --- | --- | --- | --- | --- |
| Untested: Re-hospitalization | 0.70 | $11,200 | $7,840 |  |
| Tested: Re-hospitalization | 0.33 | $11,200 | $3,696 | $4,144 |
| Untested: ED Visits | 0.66 | $884 | $543 |  |
| Tested: ED Visits | 0.39 | $884 | $345 | $238 |
| **Total per patient savings in 60-days prior to cost of intervention** | | | | $4,382 |

Medicare average all-cause readmission cost [32] in 2009 was $11,200 and average ER visit cost [33] in 2011 was $884. Model based on Medicare average showed a $4382 per patient cost savings in 60 days balanced by a test cost of $914. ED, Emergency Department; ER, Emergency Room.
